# Advanced Lung-on-a-Chip Technology: Mimicking the Complex Human Lung Microenvironment

**DOI:** 10.7150/ijbs.105702

**Published:** 2025-01-01

**Authors:** Eun-Kyung Min, Choon-Mi Lee, Soo-Rim Kim, Jin Woo Lee, Chan Hum Park, Byung-Chul Oh, YunJae Jung, Hwa-Yong Lee

**Affiliations:** 1 Department of Health Sciences and Technology, GAIHST, Gachon University, Incheon 21999, Republic of Korea.; 2 Department of Molecular Medicine, School of Medicine, Gachon University, Incheon 406-840, Republic of Korea.; 3 Department of Otolaryngology-Head and Neck Surgery, Chuncheon Sacred Heart Hospital, Hallym University College of Medicine, Chuncheon, Republic of Korea.; 4 Department of Physiology, Lee Gil Ya Cancer and Diabetes Institute, Gachon University College of Medicine, Incheon, 21999, Republic of Korea.; 5 Department of Microbiology, College of Medicine, Gachon University, Incheon 21999, Korea.; 6 Division of Science Education, Kangwon National University, 24341, Republic of Korea.

**Keywords:** Lung-on-a-Chip, Lung Toxicity, SERPINB2, Natural Polymers, Fluorescence Screening

## Abstract

Intricate crosstalk among various lung cell types is crucial for orchestrating diverse physiological processes. Traditional two-dimensional and recent three-dimensional (3D) assay platforms fail to precisely replicate these complex communications. Many *in vitro* lung models do not effectively reflect the multicellular complexity of lung tissue. Here, we fabricated an advanced multicellular 3D lung-on-a-chip system that properly replicates the dynamic pulmonary microenvironment and its intricate microarchitecture. Diverse lung cells were incorporated into a microstructure formed from a mixture of natural polymers, including collagen and hyaluronic acid, and blood coagulation factors acting as natural crosslinking agents. The system accurately reflects the complex 3D architecture of the lung. Biomarkers demonstrate more rapid and sensitive responses to toxic substances than functional indicators, such as cell proliferation and apoptosis. SERPINB2 was identified as a biomarker of lung toxicity; it was activated in small airway epithelial cells exposed to various toxic substances. We then developed a fluorescence-linked toxicity biomarker screening platform that enables both intuitive and quantitative evaluation of lung toxicity by measuring the converted fluorescent signal strength. This fluorescent tagging system was incorporated into small airway epithelial cells within a fabricated chip platform; enabling lung-on-a-chip enabled evaluation of the lung toxicity of prospective drug candidates.

## Introduction

Animal models and traditional monolayer cell culture systems fail to accurately replicate *in vivo* outcomes 1. With the goal of achieving a more comprehensive understanding of physiologically relevant tissue environments and functions, advanced three-dimensional (3D) lung-on-a-chip platforms have been developed recently 2-4. This significant advancement in 3D cell culture research remains inadequate in replicating the fundamental lung architecture and intricate spatial arrangements of the diverse pulmonary cell types. Current lung-on-a-chip systems are also unable to replicate the complex reciprocal cellular crosstalk between various lung cells that is essential in maintaining pulmonary homeostasis and respiratory function. Therefore, to develop a multi-compartmentalized lung-on-a-chip platform made of polydimethylsiloxane (PDMS), we employed computer-aided design (CAD) software and 3D printing techniques to design a compartmentalized microchannel architecture consisting of a respiratory airway and lateral supporting stromal chambers separated by a thin porous barrier on which vascular endothelial cells were incorporated.

The physiological relevance of nearly all human lung-on-a-chip systems is challenged by the use of lung cancer cell lines that often fail to accurately replicate the physiology of normal lung alveolar epithelial cells 5-7 or primary pulmonary cells derived from rodents that are compromised by interspecies discrepancies that make them unsuitable for accurately replicating the organ-level functionalities of the human lung 8, 9. Therefore, to reflect the 3D pulmonary microenvironment, we incorporated human small airway epithelial cells into the respiratory airway chambers of a lung-on-a-chip platform using a mixture of natural polymers (hyaluronic acid and collagen). Small airway epithelial cells are crucial in maintaining the functionality and physiological characteristics of the lung 10. These cells form a protective barrier lining the respiratory tract that is first line of defense against inhaled pathogens, particulate matter, and environmental toxins 11. They are actively involved in mucociliary clearance, which is vital for trapping and expelling foreign particles and pathogens from the airways through the coordinated action of cilia and mucus production 12. In addition, human stromal cells (fibroblasts) and macrophages containing the natural polymer mixture were incorporated into the supporting stromal chambers of the lung-on-a-chip platform. These two chambers are surrounded by vascular cell-coated media channels, facilitating reciprocal crosstalk between various loaded lung cell types through the exchange of diverse growth factors, cytokines, and immunomodulatory factors.

Despite the excellent biocompatibility of natural polymer-derived tissue architectures within the chip system, their structural integrity is inadequate for replicating the structural properties of lung tissue because of their inability to form covalent crosslinks that impart the necessary mechanical strength 13. Several chemical crosslinkers have been used to enhance the physical strength and structural integrity of natural polymer tissue constructs 14. However, a major drawback of chemical crosslinking methods is the possible toxic effects of the remaining unreacted crosslinkers 15, which restricts their clinical utilization. Thus, we employed non-toxic proteins involved in the coagulation of blood, such as thrombin and fibrinogen, to reinforce the structural strength of natural polymer-based tissue constructs within the chip system and ensure the absence of residual toxic effects.

Early alterations in specific signaling pathways or gene expression induced by toxic exposure are more closely linked to the initial onset of toxic effects than to terminal functional outcomes, such as apoptosis and cell cycle arrest, which occur at a greater threshold 16. Therefore, evaluating these early alterations is a more efficient and precise strategy for rapidly obtaining toxicity-related information on specific drug candidates 17. Accordingly, we developed an advanced lung toxicity biomarker-based screening platform. Importantly, we identified plasminogen activator inhibitor type 2 (SERPINB2) as a reliable indicator of lung toxicity, including apoptosis and cell cycle arrest, in human small airway epithelial cells. The gene encoding green fluorescent protein (GFP) was integrated into the regulatory sequence of SERPINB2, enabling both the intuitive and quantitative evaluation of biomarker-conjugated lung toxicity. This GFP-conjugated detection system was subsequently integrated into small airway epithelial cells embedded within the corresponding respiratory airway chamber of the fabricated chip.

To the best of our knowledge, this is the first study to develop a multicellular and multi-compartmentalized lung-on-a-chip integrated with a fluorescent detection system. This lung-on-a-chip provides accurate and fast toxicity data concerning potential drug candidates whose harmful effects are rarely identified by conventional monolayer single-cell models and even advanced 3D drug evaluation systems.

## Materials and Methods

### Isolation and establishment of human lung tissue constituent cells

Normal lung tissues were meticulously collected from the surgically resected right upper lobes of patients undergoing lobectomy for lung cancer at Gachon University Gil Medical Center. Written informed consent was obtained from each patient. The study was approved by the Gachon University Institutional Review Board (Institutional Review Board No: GCIRB2019-334). The resected lung tissues were mechanically minced into small fragments, which were then subjected to enzymatic digestion in Dulbecco's modified Eagle's medium (DMEM) containing 10% fetal bovine serum (FBS) and 250 U/ml type I collagenase for 5 h at 37°C in a rotating shaker. Following digestion, the solution was filtered through a 70 µm mesh cell strainer to remove the undigested tissue fragments, followed by an additional filtration through a 40 µm mesh cell strainer to isolate the lung stromal cell populations from the vascular cells and their aggregates. Isolated lung stromal cells were cultured in high-glucose DMEM (Cat. No.: LM001-05; WELGENE Biotech, Taipei, Taiwan) supplemented with 10% FBS) and 1% penicillin/streptomycin at 37°C in an atmosphere of 5% CO_2_. Human small airway epithelial cells were obtained from PromoCell (C-12642; Heidelberg, Germany) and expanded in Small Airway Epithelial Cell Growth Basal Medium (SAGM, Cat. No.: CC-4124; No.: CC-3119; Lonza, Basal, Switzerland) supplemented with SAGM (Cat. No.: CC-4124) and 10% FBS. Normal human macrophages were obtained from ATCC (CRL-9855) and then cultured in RPMI 1640 medium containing 10% FBS and 1% Penicillin/Streptomycin. Normal HUVECs (PCS-100-010 ™), were sourced from ATCC and then maintained in Endothelial Cell Growth Basal Medium-2 (Lonza) supplemented with 10% serum and Endothelial Growth Medium-2 growth supplements. The culture medium for these cells was refreshed every 2 days to maintain optimal growth conditions. However, normal human cells exhibit limited proliferative capacity *in vitro* 18. To overcome this limitation, the cells were immortalized by stable transfection with the SV40 large T antigen, which minimally altered their original characteristics 19. These immortalized cells derived from a single clone provide a more homogeneous population than the heterogeneous nature of primary cultured cells, which often display varied morphologies.

### Construction of lung-on-a-chip platform by injection of PDMS into a 3D-printed casting mold

**Figure 1:**
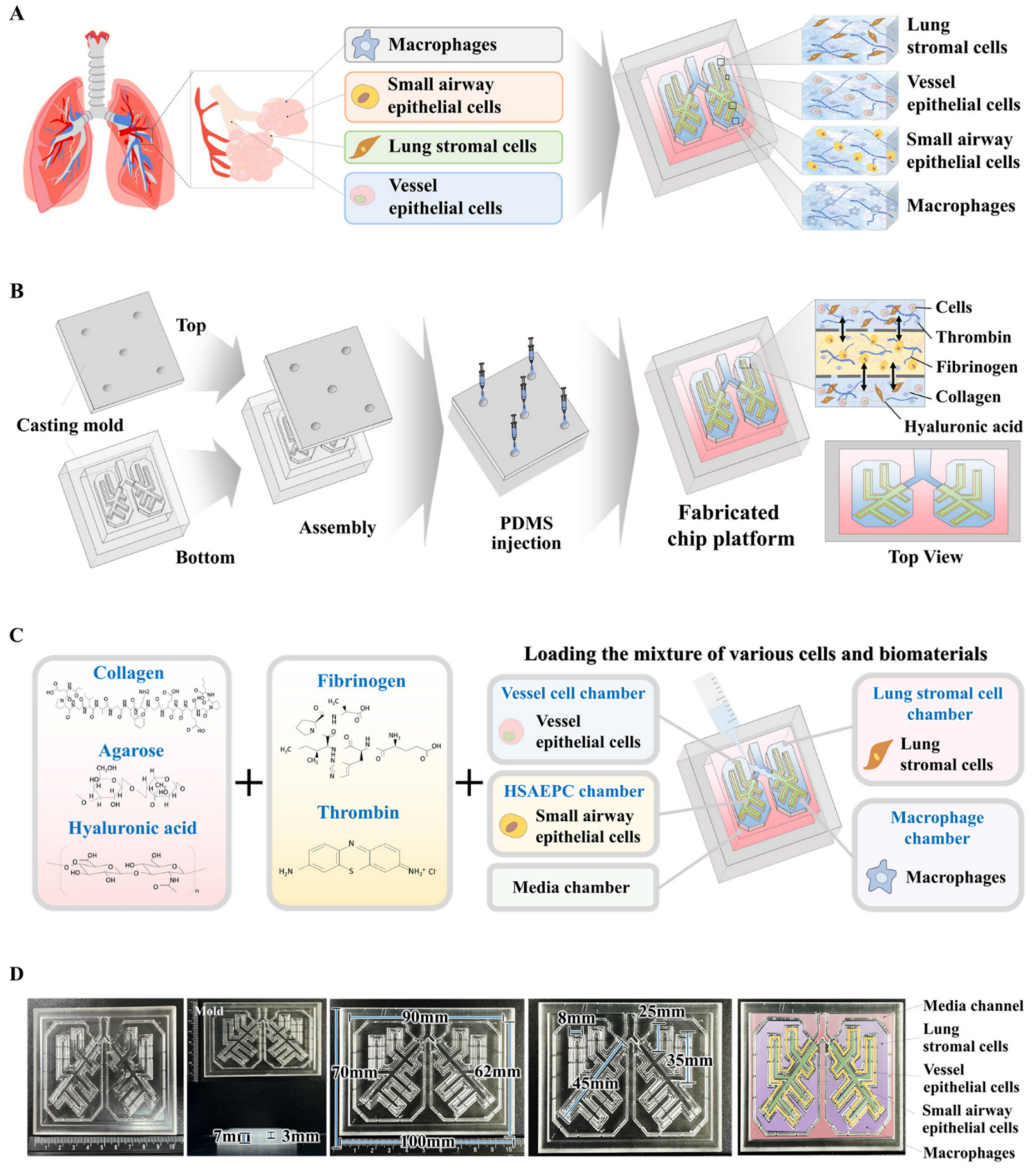
** Basic concept of human lung-on-a-chip that accurately mimics structural and multicellular complexities of lung tissue.** The figure details the core concept of the multi-compartmentalized lung-on-a-chip platform, illustrating the interactive multicellular communication between the respiratory airways and the adjacent supporting stromal chambers. This platform integrates various cellular elements and 3D structural complexities composed of natural polymers. A thin porous barrier embedded with vascular endothelial cells links the respiratory airway chamber with the supporting stromal chambers, enabling bidirectional cellular communication through the exchange of growth factors and cytokines. Furthermore, to accurately mimic the intricate multicellular interactions within lung tissue microenvironments, the two chambers are encompassed by media channels lined with human vascular cells **(A)**. The 3D mold for the human lung-on-a-chip system, designed to accurately replicate the structural characteristics of the lung tissue. The mold was produced using PLA-based 3D printing technology. Polydimethylsiloxane (PDMS) was subsequently injected into the 3D printed mold, and the resulting chip platform was extracted following polymerization process **(B)**. To replicate the physiological characteristics and multicellular diversity of lung tissue, the PDMS-based lung-on-a-chip platform was populated with multiple human lung cell types, including small airway epithelial cells, stromal cells, vascular endothelial cells, and macrophages. These cells were combined with a natural polymer mixture (hyaluronic acid and collagen), along with blood coagulating factors (thrombin and fibrinogen). The spindle-shaped respiratory airway chamber, which spans the entire chip, is populated with human small airway epithelial cells. This airway chamber is encircled by adjacent stromal chambers that house human stromal cells and macrophages, facilitating a comprehensive representation of the lung tissue microenvironment **(C)**. The PDMS-based lung-on-a-chip platform was designed in a rectangular shape, measuring 100 mm in length, 70 mm in central diameter, and 7 mm in height. The chip platform was fabricated to replicate the microenvironment of the lung tissue and facilitate multicellular communication between the respiratory airways and adjacent supporting stromal chambers** (D)**.

As depicted in Fig. 1B, a casting mold design for the lung-on-a-chip was created using CAD software. Upon completion, the 3D CAD data were converted to 3D surface geometric data consisting of various vectors types, which were then segmented along the Z-axis with a thickness of 50 μm. The information for each segmented layer was subsequently transferred to a Master EV digital light processing-based 3D printer (Carima, Seoul, Korea). The casting mold for the lung-on-a-chip was fabricated from poly(lactic) acid (PLA) using a layer-by-layer manufacturing process.

### Fabrication procedure for the PDMS-based lung-on-a-chip platform

To manufacture the lung-on-a-chip platform, PDMS (Sylgard® 184, Dow Corning Corp., Auburn, MI, USA) was prepared by mixing base and curing agent at a ratio of 20:3. The mixture was thoroughly blended for 10 min at room temperature to ensure homogeneous polymerization. The resulting PDMS solution was then injected into a 3D-printed casting mold specifically designed for lung-on-a-chip. The mold was placed in a vacuum chamber to eliminate any dissolved gases. Following degassing, the PDMS-injected mold was cured in an oven at 65°C for 24 h. Once the polymerization was complete, the mold was allowed to cool to room temperature. Finally, the fabricated chip was carefully released from the casting mold using a precision razor blade to ensure that the chip platform remained intact and undamaged.

### Incorporating diverse types of lung tissue cells in each segment of the constructed chip system

To embed various lung tissue constituent cells within the compartments of the fabricated lung-on-a-chip platform, the following process was employed. Initially, solutions of type I collagen (3 mg/ml) and hyaluronic acid (3 mg/ml) were prepared in DMEM. To enhance the mechanical strength, a natural polymer mixture containing collagen and hyaluronic acid, along with blood coagulation factor solutions of 12.5 mg/ml fibrinogen and 1.25 U/ml thrombin was also prepared in DMEM. Human small airway epithelial cells were cultured in SABM; Cat. No.: CC-3119) supplemented with SAGM (Cat. No.: CC-4124). Vascular endothelial, immune (macrophages), stromal, and small airway epithelial cells were reconstituted in a balanced 1:1:1:1 mixture of hyaluronic acid (24 mg/ml), collagen (24 mg/ml), fibrinogen (50 mg/ml), and thrombin (5 U/ml). This cell-polymer mixture was loaded into each compartment of the PDMS-based lung-on-a-chip platform using a syringe, without the need for additional ECM coating. The mixture was allowed to polymerize by cooling to room temperature for 30 min. An elongated respiratory airway chamber with a diameter of 3 mm and thickness of 8 mm was formed with cell densities of approximately 2 × 10^5^ cells/ml within the tissue construct. The respiratory airway chamber was surrounded by supporting stromal chambers that house the human stromal cells and macrophages. Consistency was maintained across both the respiratory airway and supporting stromal chambers using a uniform ratio of a natural polymer complex comprising type I collagen and hyaluronic acid, along with a fibrinogen-thrombin mixture. The culture medium was refreshed every two to three days to sustain cell viability and function.

### Evaluation of microstructure, compressive strength, and rheological properties of the produced lung tissue constructs

The microstructural characteristics of the natural polymer-based lung tissue architectures were examined using variable pressure and field emission SEM (EVO®LS10; Carl Zeiss, Jena, Germany) at the Korean Basic Science Institute in Chuncheon, Korea. Fabricated lung tissue samples were freeze-dried and coated with a thin 10 nm layer of gold/palladium for 30 s at a discharge current of 15 mA using an Ion Sputter 1010 device (Hitachi, Tokyo, Japan). Microstructural images were captured at an accelerating voltage of 1.2 to 1.3 kV following established protocols 20.

The compressive stress-strain behavior of the lung tissue architecture was evaluated using a model QM100S universal testing machine (QMESYS, Gunpo, Korea). Cylindrical samples of natural polymer-based lung tissue architectures, each with a diameter of 10 mm and a height of 3 mm, were subjected to uniaxial compression tests. A gradual compression force was applied at a displacement rate of 5 mm/min until the samples fractured, allowing for the calculation of the stress at failure in accordance with previously documented methods 20.

Concerning rheological properties, the viscosity of the fabricated tissue architecture was assessed at 37°C using a model MCR 102 rheometer (Anton Paar, Zofingen, Switzerland). The shear rate was varied from 1 to 20 s^-1^ to evaluate potential shear thinning or thickening behavior. This analysis was performed following established protocols to ensure consistency and reliability of the results 20.

### Evaluation of extended cell viability of embedded cells in the lung-on-a-chip

The prolonged viability of cells embedded within the constructed lung-on-a-chip platform was evaluated using a Live & Dead assay (Cat. No: L3224; Invitrogen, Carlsbad, CA, USA) 1, 7, 14, 21, and 28 days post embedding, according to the manufacturer's instructions. To prepare for the assay, each compartment of the chip was rinsed three times with serum-free DMEM. One milliliter of the assay solution containing 2 mM ethidium homodimer-1 and 4 mM calcein AM was introduced into each chamber. Following a 30-min incubation at room temperature, the compartments were examined using the EVOS FL Cell Imaging System (Thermo Fisher Scientific, Waltham, MA, USA) to assess cell viability.

### Assessment of prolonged metabolic activities of embedded cells in the lung-on-a-chip

The extended metabolic activities of the embedded cells within the lung-on-a-chip were evaluated using the CCK-8 assay (Cat. No: KTC011001; Abbkine, Atlanta, GA, USA) according to the manufacturer's instructions. For the assay, each compartment was treated with 100 µl/ml of CCK-8 solution in serum-free DMEM. The chip containing the cells was then incubated for 4 h at 37°C in a 5% CO_2_ incubator. Metabolic activity was quantified by measuring the absorbance at 450 nm using a SoftMax Pro 5 microplate reader (Molecular Devices, San Jose, CA, USA).

### Antibody-based fluorescence staining of embedded cells in the lung-on-a-chip

For fluorescence staining, samples were fixed with 4% paraformaldehyde. They were then permeabilized using a solution containing 0.4 M glycine and 0.3% Triton X-100. To prevent nonspecific binding, the samples were blocked with 2% normal swine serum (DAKO, Glostrup, Denmark). Staining was performed as previously detailed 21 using primary antibodies to GFP (Cat. No: V820-20; Invitrogen), and PECAM1 (Cat. No.: BBA7; R&D Systems, Minneapolis, MN, USA), vWF (Cat. No.: ab6994; Abcam, Cambridge, UK), vimentin (Cat. No.: 550513; BD Biosciences, Santa Clara, CA, USA), fibronectin (Cat. No.: ab2413; Abcam), cytokeratin 18 (Cat. No.: sc-32329; Santa Cruz Biotechnology, Dallas, TX, USA), cytokeratin 19 (Cat. No.: sc-376126; Santa Cruz Biotechnology), CD68 (Cat. No.: sc-20060; Santa Cruz Biotechnology), CD11b (Cat. No.: ab52478; Abcam), COL1A1 (Cat. No.: PA5-29569; Invitrogen), laminin α1 (Cat. No.: PA1-16730; Invitrogen), MUC5AC (Cat. no.: ab198294; Abcam), MUC5B (Cat. No.: 37-7400; Invitrogen), and SPDEF (Cat. No.: BS-1866R; Bioss, Woburn, MA, USA). The expression patterns of these proteins were visualized and analyzed by fluorescence microscopy using a model LSM 510 Meta instrument (Carl Zeiss).

### Real-time PCR analysis

Total RNA was extracted using TRIzol reagent (Invitrogen Life Technologies, Carlsbad, CA, USA) following the manufacturer's protocol. The purity of isolated RNA was confirmed by assessing the 260/280 nm absorbance ratio. First-strand cDNA synthesis was conducted from 1 μg of total RNA using SuperScript II (Invitrogen Life Technologies). Subsequently, 10% of the synthesized cDNA was used in each PCR mixture, which included Express SYBR Green qPCR Supermix (BioPrince, Seoul, South Korea). Real-time PCR was performed using a Rotor-Gene Q real-time PCR cycler (QIAGEN, Valencia, CA, USA). The PCR conditions involved 40 cycles of denaturation at 95°C for 20 s, annealing at 60°C for 20 s, and extension at 72°C for 25 s. The relative mRNA expression levels of the target genes were normalized to the expression of peptidylprolyl isomerase A and calculated using the ΔΔCT method. The primer sequences used for PCR are listed in Table 1.

### Protein extraction and immunoblotting

The cells were lysed in a buffer composed of 50 mM Tris, 5 mM EDTA, 150 mM NaCl, 1 mM dithiothreitol, 0.01% NP-40, and 0.2 mM phenylmethylsulfonyl fluoride. Protein concentrations in the lysates were determined using bovine serum albumin solutions of known concentrations as standards. Equal amounts of protein from each sample were separated based on molecular weight by sodium dodecyl sulfate-polyacrylamide gel electrophoresis. Following electrophoresis, proteins were transferred to nitrocellulose membranes (Bio-Rad, Hercules, CA, USA) via electrophoretic transfer. The membranes were blocked with 5% (w/v) nonfat dry milk in Tris-buffered saline containing Tween-20 (TBS-T) for 1 h at room temperature. Subsequently, the membranes were incubated overnight at 4°C with primary antibodies to SERPINB2 (Cat. No: ab47742; Abcam), MMP-2 (Cat. #4022; Cell Signaling Technology, Beverly, MA, USA), MMP-9 (Cat. #13667; Cell Signaling Technology), caspase-3 (Cat No. #9662; Cell Signaling Technology), and β-actin (Cat No.: ab189073; Abcam). After incubation with the primary antibody, the membranes were washed and incubated with horseradish peroxidase-conjugated secondary antibodies, including goat anti-rabbit IgG (554021; BD Pharmingen, San Diego, CA, USA) and goat anti-mouse IgG (554002; BD Pharmingen), for 60 min at room temperature. The bound antibodies were visualized using an enhanced chemiluminescence reagent.

### Transwell invasion/migration experiment

To evaluate cell migration, cells (1 × 10^5^ per well) were placed in the upper chambers of a Transwell plate (Corning Inc., Corning, NY, USA), which featured 8.0 μm pores within 6.5 mm-diameter polycarbonate membranes. The Transwell system was arranged in a 24-well plate. Nonmigrating cells remaining on the upper surface of the membrane were removed by gentle scrubbing with laboratory paper. The cells that successfully migrated to the lower side of the membrane were fixed with 4% paraformaldehyde for 5 min and stained with hematoxylin for 15 min. The numbers of migrating and invading cells were counted in three randomly selected fields per well using an optical microscope at 50× magnification.

### ELISA

Fibronectin secretion assay (Cat. No.: ELH-FN1; RayBiotech, Peachtree Corners, GA, USA), and laminin (Cat. No.: ELH-LAMA4; RayBiotech) from the lung stromal cell-loaded chamber was assessed using ELISA kits following the manufacturer's protocols. The optical density of each well was measured at 450 nm using a microplate reader. The fibronectin and laminin concentrations in each sample were calculated using a standard curve. All experiments were conducted in triplicate, and each sample was analyzed in duplicate.

### Targeted suppression of SERPINB2 via specific shRNAs

To achieve targeted SERPINB2 knockdown, specific shRNA (accession no. NM_002575) and scrambled control shRNA were obtained from Bioneer (Daejeon, South Korea). Transfections were performed using Lipofectamine 2000 (Cat. No. 52887; Invitrogen) according to the manufacturer's protocol. The shRNA targeting SERPINB2 (3 μg/ml) was combined with 3 μl of Lipofectamine 2000 in serum- and antibiotic-free Opti-MEM (Gibco, Frankline Lakes, NJ, USA). Five hours before transfection, the Opti-MEM was replaced with fresh human Small Airway Epithelial Cell Growth Basal Medium supplemented with SAGM Single Quots and 10% serum. The SERPINB2 shRNA construct, optimized for high transfection efficiency at the mRNA level, was used for gene silencing.

### Examination of GEO datasets

The GEO (https://www.ncbi.nlm.nih.gov/geo/) is an extensive international repository for wide-ranging gene expression datasets obtained from RNA-seq, DNA microarrays, and chip sequencing 22, 23. The GEO datasets were systematically organized into four primary sections: experimental designs, raw data, groups, and platforms. The results from each dataset were further classified based on several factors, including treatment conditions, physiological conditions, and disease states. These categorized data are displayed as "GEO profiles," which feature rank measurements, functional annotations, and charts showing expression values, for each gene across all analyzed samples 24. To assess the expression profile of SERPINB2 in response to exposure to various toxic materials, the data were analyzed according to established protocols 24.

### Statistical evaluation

All statistical analyses were performed using GraphPad Prism 9.0 (GraphPad Software, San Diego, CA, USA). A two-tailed Student's t-test was used to evaluate the data. Statistical significance was set at P < 0.05.

## Results

### Synthesis of a multi-compartmentalized lung-on-a-chip system that properly mimics structural and multicellular complexity, and reciprocal interactions

The multi-compartmentalized lung-on-a-chip platform is detailed in Fig. 1. The interactive multicellular communication between the respiratory airways and adjacent supporting stromal chambers, and incorporated multicellular elements and 3D structural complexities made of natural polymers are illustrated. A thin porous barrier embedded with vascular endothelial cells directionally interconnects the respiratory airway and the adjacent supporting stromal chambers. This structure facilitates reciprocal cellular communication between the two chambers through the exchange of various growth factors and cytokines. Additionally, to properly replicate the complex multicellular interactions within the lung tissue microenvironment, the two chambers are surrounded by media channels coated with human vascular cells (Fig. 1A).

Lung-on-a-chip synthesis was performed in several successive stages. To consistently and reliably produce an lung-on-a-chip platform capable of sustaining the multi-compartmentalized structural complexities of lung tissue, a casting mold for the chip body was created using polylactic acid (PLA)-based 3D printing techniques. PLA was chosen because of its exceptional printability, which allows for precise and complex designs, and robust mechanical properties that provide durability and structural integrity 25. Subsequently, polydimethylsiloxane (PDMS) was injected into the 3D printed mold and allowed to polymerize at room temperature. After polymerization, the multi-compartmentalized lung-on-a-chip platform was carefully removed from the casting mold (Fig. 1B). An elongated 3 mm-diameter respiratory airway chamber loaded with spindle-shaped human small airway epithelial cells was extended across the entire fabricated, rectangular, PDMS-based lung-on-a-chip platform (70 × 100 × 7 mm^3^). The arrangement closely mimicked the bronchial structure of the lung. The respiratory airway chamber was surrounded by supporting stromal chambers containing human stromal cells and macrophages. The two chambers were directionally interconnected by a 2 mm-wide thin porous barrier embedded with vascular endothelial cells (Fig. 1C). Next, to replicate the multicellular complexity of the lung, various cells that are present in the lung, including small airway epithelial cells, stromal cells, vascular endothelial cells, and macrophages, were combined with a natural polymer mixture of collagen and hyaluronic acid, along with fibrinogen and thrombin, which coagulate blood and are non-toxic, to reinforce the mechanical strength of the natural polymer mixtures. This lung cell-polymer mixture was then loaded into the corresponding compartments of the multi-compartmentalized chip platform (Fig. 1D).

### Microstructure, mechanical strength, and rheological behavior of the natural polymer-based tissue scaffold in the lung-on-a-chip platform

**Figure 2:**
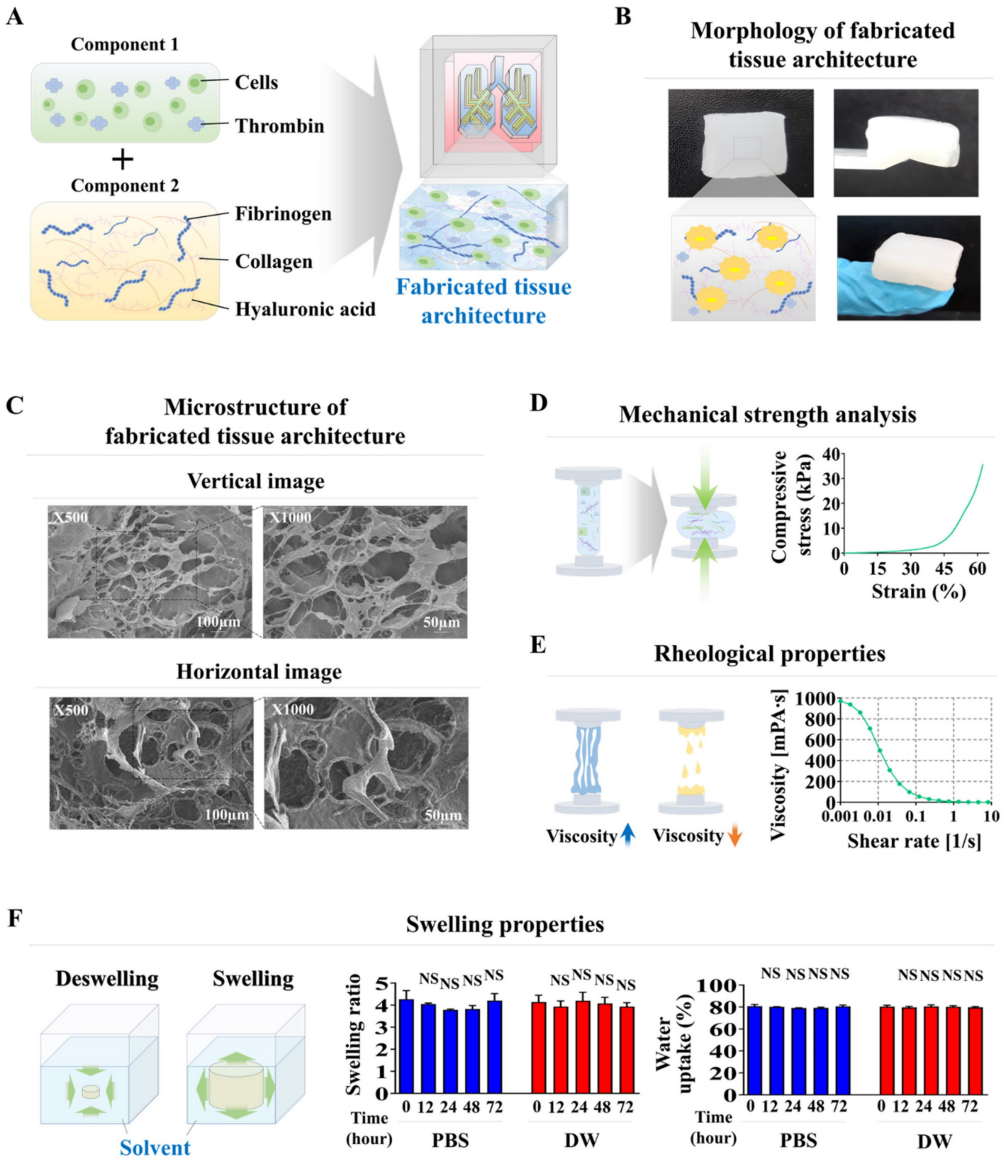
** Fabrication of natural polymers-based lung tissue constructs and examination of its physical properties.** The diverse cellular constituents constituting lung tissue were integrated with a combination of natural polymer, including hyaluronic acid and collagen, along with non-toxic blood coagulating factors thrombin and fibrinogen** (A)**. The synthesized tissue architecture exhibited a refined surface, soft texture, and white coloration **(B)**. Cross-sectional and lateral microstructural images of the tissue constructs examined by scanning electron microscopy (SEM). SEM images revealed a homogeneously dispersed porous matrix, with pores ranging from approximately 50 to 100 μm in diameter, resulting from the crosslinking of natural polymers combined with blood coagulating factors **(C)**. The mechanical characteristics of the fabricated tissue architecture were evaluated using a universal test machine by administering single-axis compression forces. The tissue samples, each measuring 10 mm in diameter and 10 mm in height, were subjected to varying levels of uniaxial compressive stress to determine their mechanical behavior. To determine the precise failure stress of the fabricated samples, uniaxial compressive force was applied at a constant loading rate of 5 mm/min until the samples fractured **(D)**. The dynamic viscosity of the fabricated tissue architecture was assessed across a range of shear rates from 1 to 10/s. The viscosity of the fabricated tissue architecture progressively decreased from approximately 1000 to 0 Pa-sec **(E)**. To evaluate the swelling dynamics of tissue structures, fabricated samples were immersed in both distilled water and PBS (pH 7.4) at 37°C for 24 h. After the hydration period, the liquid was carefully removed. The tissue architectures were subsequently weighed to assess their water uptake capacity **(F)**. All experiments were performed in triplicate. Significant differences are indicated as follows: *, *p* < 0.05; **, *p* < 0.005; and ***, *p* < 0.001 (two-sample t-test).

The combination of collagen and hyaluronic acid is widely utilized in various tissue engineering applications to replicate the tissue microenvironment because of its reliable structure, consistent uniformity, and excellent tissue compatibility 26-28. These natural polymer-based scaffolds create a structural matrix that promotes cell adherence and viability, and essential signaling pathways that regulate diverse cellular activities, including cell growth, movement, and differentiation 29, 30. However, the structural integrity of these natural polymers is insufficient to mimic the physical characteristics of various normal tissues. Therefore, to enhance their mechanical robustness without inducing cellular toxicity, blood coagulation factors that include fibrinogen and thrombin have been employed as natural polymerizing agents. Tissue scaffolds fabricated using mixtures of different combinations of blood coagulation factors (fibrinogen and thrombin) and natural polymer (collagen and hyaluronic acid) within each compartment of the chip platform displayed a smooth surface, distinct shape with clear boundaries, and soft, white appearance (Fig. 2A and 2B). The microstructure of the constructed tissue scaffold in each compartment of the chip was analyzed by scanning electron microscopy (SEM). This examination revealed a consistently distributed porous network with pore diameters ranging from approximately 50 to 100 μm throughout the scaffold (Fig. 2C). An intricate microporous microstructure developed through the stable polymerization of hyaluronic acid and collagen fibers, facilitated by blood coagulation factors, with no cytotoxicity. This porous structure enhances cell attachment and survival rates 31. A significant challenge for natural polymer-based tissue scaffolds is maintaining sufficient mechanical strength to maintain their structural integrity, which is necessary to support cell survival and attachment of incorporated cells 32, 33. The mechanical strength of the natural polymer-based scaffold fabricated in the present study was approximately 40 kPa, consistent with the previously reported mechanical strength of soft human lung tissue of approximately 50 kPa 34 (Fig. 2D). The rheological property of viscosity is crucial to the performance of biomaterial-based scaffolds; viscosity significantly affects the viability of the incorporated cells 35. For example, a natural and low viscosity polymer-based tissue scaffold can effectively maintain its structural integrity but typically results in reduced viability of embedded cells 36. The rheological properties of fabricated tissue scaffolds were evaluated by examining their dynamic viscosities at various shear rates. As the shear rate increased from 1/s to 10/s, the rheological properties of the fabricated tissue scaffold progressively deteriorated from approximately 1000 to 0 Pa s (Fig. 2E). Evaluations of the swelling potential of fabricated tissue scaffolds in both distilled water and PBS at 37°C and pH 7.4 showed that their structural integrity and volume remained largely unchanged upon water absorption (Fig. 2F). These findings indicate that our fabricated tissue scaffold possesses appropriate physical characteristics for incorporating various cells from lung tissue.

### Cellular compatibility evaluations of distribution patterns, viabilities, metabolic functions, and molecular profiles of various lung cells integrated within the lung-on-a-chip

Although fabricated natural polymer-based scaffolds possess optimal physical properties for cell encapsulation, various technical obstacles remain to be resolved. These include ensuring even distribution and long-term cell viability of the incorporated cells, preserving their cellular properties, and performing appropriate functions within the tissue scaffold. Accordingly, we examined the distribution patterns, cell viability, metabolic functions, and molecular profiles of various lung cells incorporated into a natural polymer-based scaffold. Four distinct lung cell types were incorporated in the chip system chamber. Human lung stromal cells were freshly isolated from normal lung tissue of patients undergoing either pneumonectomy or lobectomy for cancer treatment (Supplemental Fig. 1A). These cells were identified by their spindle-shaped morphology and notable self-renewal ability (Supplementary Fig. 1B). Fibronectin 37 and vimentin 38 produced by lung stromal cells (fibroblasts) modify cell autophagy and significantly enhance cell invasiveness. Immunostaining results clearly demonstrated that these cells were strongly positive for established lung stromal cell biomarkers (Supplementary Fig. 1C). Small airway epithelial cells were also morphologically characterized by their polygonal shape (Supplementary Fig. 2A) and relatively high expression of specific surface biomarkers, such as cytokeratin 18 and 19 (Supplementary Fig. 2B). Human umbilical vein endothelial cells (HUVECs) have been used as a model for lung vessel cells because of their widespread application in various vascular studies 39. These cells were distinguished by their round morphology (Supplementary Fig. 3A) and positive expression of platelet endothelial cell adhesion molecule 1 (PECAM-1) and Von Willebrand factor (vWF) (Supplementary Fig. 3B). In addition, lung macrophages are crucial for maintaining homeostasis, clearing pathogens, and regulating immune responses 40. Consequently, human macrophages were used as a cellular model for immune cells residing in lung tissue. These cells were defined by their characteristic round shape (Supplementary Fig. 4A) and expression of specific surface markers, including CD11b and CD68 (Supplementary Fig. 4B).

**Figure 3:**
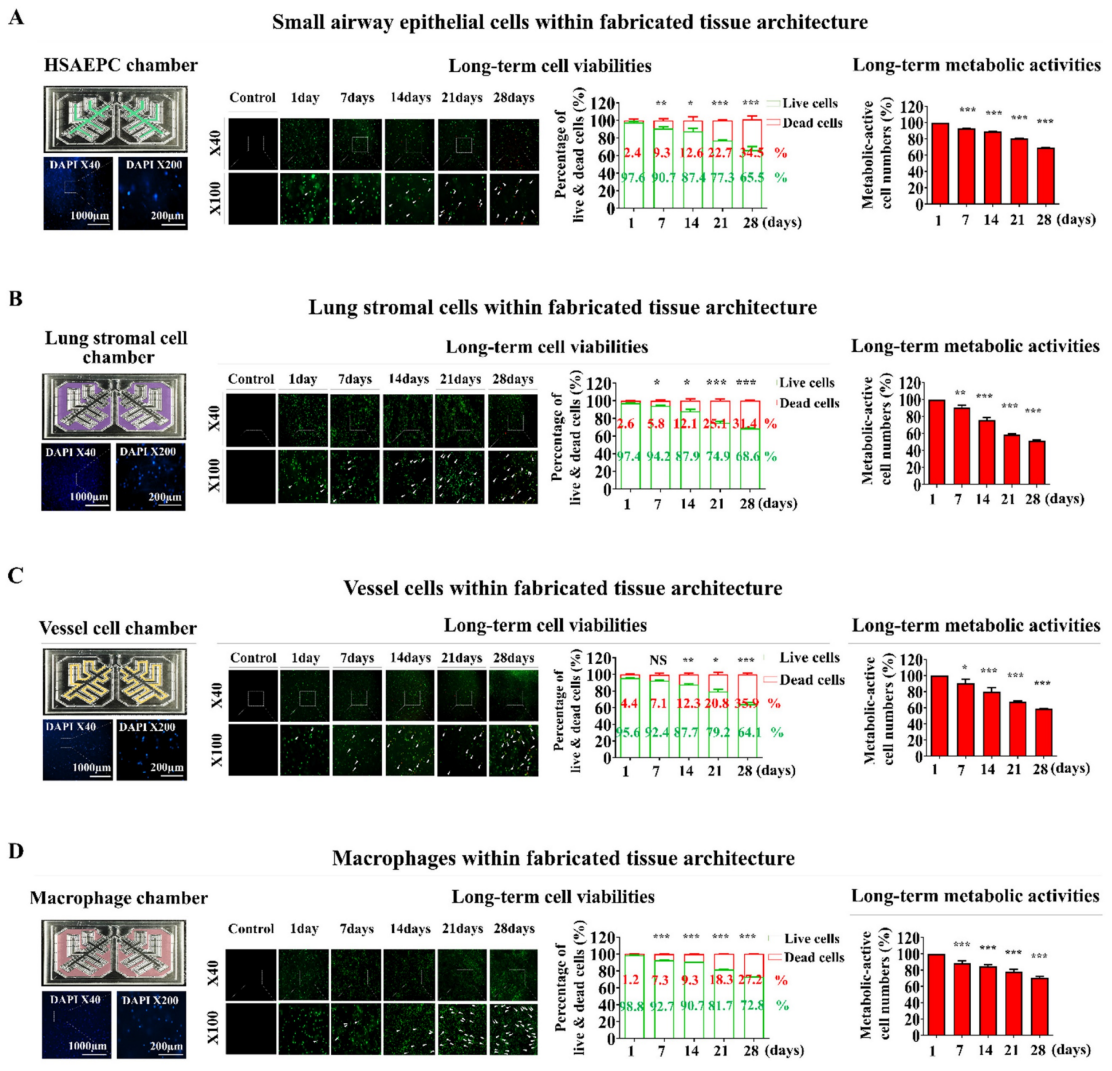
** Examination of the sustained viability and metabolic functions of multiple cells embedded within each segment of the lung-on-a-chip model.** The spatial arrangement of diverse cells integrated into each section of the chip was investigated by first cultivating the tissue structures in a cell-specific culture medium for 24 h, followed by staining with the DNA-targeting fluorochrome 4′,6-diamidino-2-phenylindole (DAPI). The patterns of cellular distribution across each tissue structure were assessed with by fluorescent microscopy. Different types of lung tissue cells are integrated within each compartment of the lung-on-a-chip, including small airway epithelial cells **(A)**, stromal cells **(B)**, vascular endothelial cells **(C)**, and macrophages **(D)**. Following this, the cells were cultured in specialized medium tailored for each cell type and incubated for 1, 7, 14, 21, or 28 days post-integration. Cell viability was assayed using the live & death analysis, using fluorescent dyes to distinctly mark live (green) and dead (red) cells. The prolonged viability of cells in each chamber was then assessed by fluorescent imaging. Seventy or greater percent of the cells embedded in each compartment of the chip remained viable for 28 days, as indicated by the aforementioned green and red fluorescence. Additionally, each compartment of the chip was incubated in a serum-free setting with CCK-8 solution for 48 h. The metabolic functions of the integrated cells were then evaluated by determining the optical density at 450 nm. All experiments were performed in triplicate. Significant differences are indicated as *, *p* < 0.05; **, *p* < 0.005; and ***, *p* < 0.001 (two-sample t-test).

These lung tissue cells were incorporated into a natural polymer mixture of hyaluronic acid and collagen, along with the thrombin and fibrinogen blood coagulation proteins. They were carefully embedded in each compartment of the lung-on-a-chip platform. Nuclear staining was used to analyze the distribution profiles within the chip platform. Ensuring even distribution of integrated cells throughout the chip is essential for preserving organ-specific functions and achieving consistent and precise test outcomes 31. The incorporated cells were homogenously and uniformly dispersed across the entire natural polymer-based tissue scaffold within the chip system (Fig. 3A-D). Cell viability was also assessed at multiple times to evaluate the prolonged viability of the integrated cells within the lung-on-a-chip platform. Cell survival rates gradually declined. Nonetheless, the majority of the incorporated cells (>87%) were still viable after 14 days, and approximately 65% remained alive after 28 days (Fig. 3A-D). The prolonged metabolic functions of integrated cells within the lung-on-a-chip were also analyzed weekly following cell incorporation using Cell Counting Kit-8 (CCK-8) assays. While the sustained metabolic functions of various embedded cells gradually declined over time, approximately 70% of the incorporated cells retained their metabolic functions after 21 days within the chip system (Fig. 3A-D).

**Figure 4:**
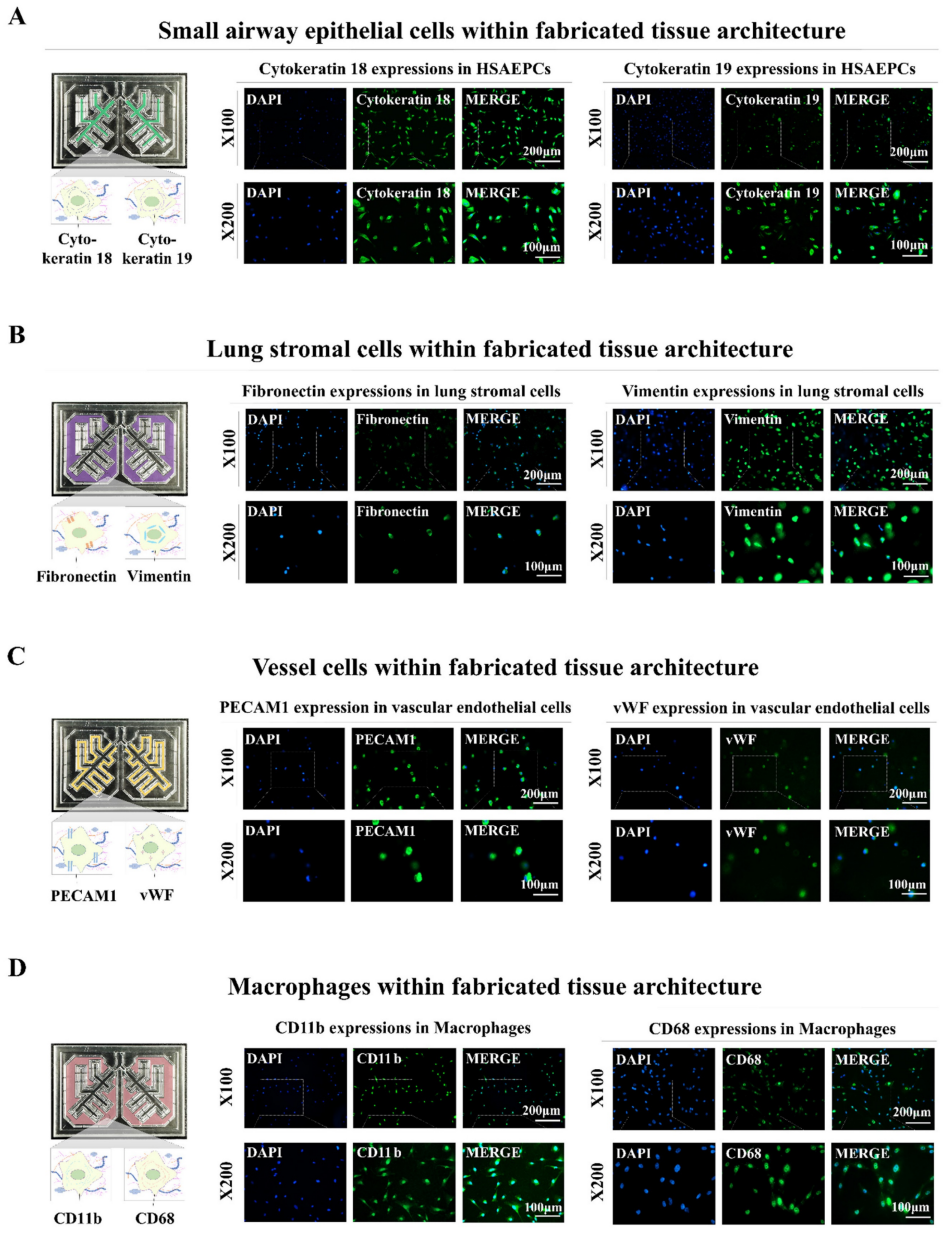
** Preservation of distinct cellular characteristics in the natural polymer-based tissue architecture.** An assessment was performed to determine if different cells retained their molecular properties following integration into each compartment of the chip. This involved cultivating the cells in tailored medium for a week and subsequently examining them with established biomarkers specific to each cell type. Staining was performed on human small airway epithelial cells for cytokeratin 18 and 19 **(A)**, while lung stromal cells were examined for fibronectin and vimentin **(B)**. Additionally, vascular endothelial cells were labeled for PECAM1 and vWF **(C)**, and macrophages were analyzed using CD11b and CD68 **(D)**. Each experiment was conducted three times. The nuclei in each field were stained with DAPI.

In addition, distinct biomarkers were used to analyze the incorporated cells to ascertain whether they could maintain their original molecular characteristics within the fabricated lung-on-a-chip platform. Cytokeratins 18 and 19, which are biomarkers of small airway epithelial cells, were abundantly expressed in the incorporated cells (Fig. 4A). Fibronectin and vimentin, which are lung stromal cell surface proteins that are crucial for migration and invasion were substantially expressed in the integrated stromal cells (Fig. 4B). Vascular endothelial cells integrated within the chip platform exhibited consistent expression of PECAM1 and vWF, which are well-established biomarkers for these cells (Fig. 4C). The incorporated human macrophages also showed substantial expression of the known biomarkers CD11b and CD68 (Fig. 4D). These findings indicate that various types of encapsulated cells can preserve their specific molecular characteristics within the biomaterial-based 3D tissue microenvironment within the chip.

### Functional analyses of the lung cell-incorporated chip platform: expression of essential proteins, secretion of key factors, and functionality of mucus-producing cells

**Figure 5:**
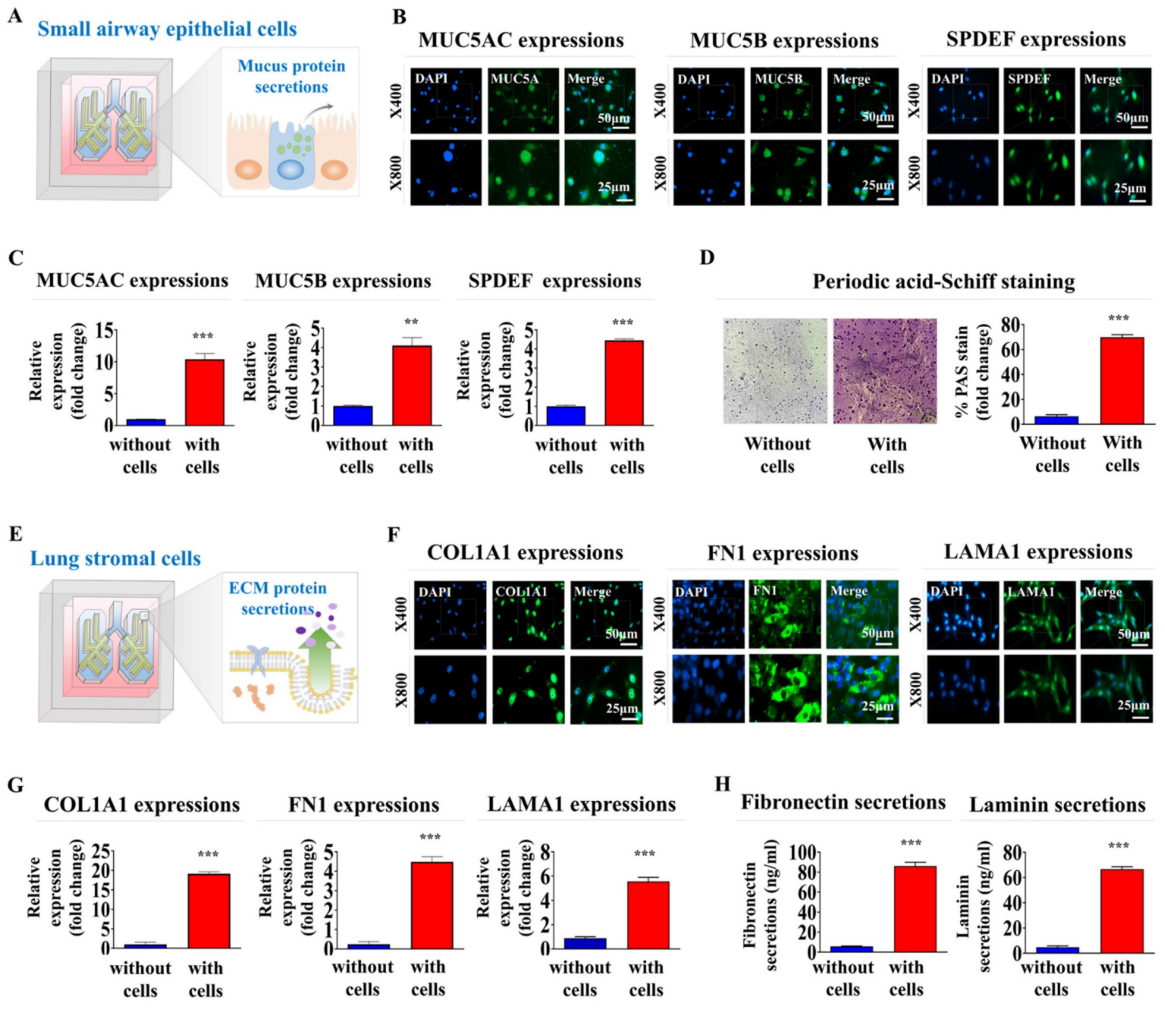
** Functional assessments of the respiratory airway and adjacent stromal chambers within the chip: critical protein expression and secretion, and mucin production.** To assess whether incorporated cells retained their distinctive properties within respiratory airway chamber in the chip, samples were cultured in a specific culture medium for 7 days before being evaluated with targeted biomarkers **(A)**. Within the natural polymer-based 3D microenvironment of the chip, human small airway epithelial cells exhibited the expression of critical proteins, including MUC5AC, MUC5B, and SPDEF, at both the protein **(B)** and mRNA **(C)** levels. Periodic acid-Schiff (PAS) staining to evaluate whether embedded human airway epithelial cells within a chip platform could properly produce glycogen, mucopolysaccharides, and other carbohydrate-rich macromolecules **(D).** To determine whether the integrated cells preserved their unique characteristics within the stromal compartment of the chip, the samples were maintained in a designated culture medium for a week and subsequently analyzed using specific biomarkers **(E)**. In the chip's 3D microenvironment constructed from natural polymers, human stromal cells showed expression levels of key proteins, such as COL1A1, fibronectin 1, and laminin α1, at the protein **(F)** and mRNA **(G)** levels. To evaluate if human stromal cells embedded in a chip platform were capable of adequately secreting essential proteins into the chamber, each cell-loaded and cell-free chamber was cultured in a specific medium tailored for the respective cell types for 7 days. Following this, the medium was switched to a serum-free variant. After 48 h of incubation, the medium was collected and tested for the presence of secreted fibronectin and laminin from the stromal chamber **(H).** All experiments were performed in triplicate. Significant differences are indicated as *p < 0.05, **p < 0.005, and ***p < 0.001 (two-sample t-test).

Mucin 5AC (MUC5AC) and MUC5B are key mucin proteins that trap and clear pathogens and particles from the airways 41. Sterile alpha motif pointed domain-containing erythrocyte transformation specific transcription factor (SPDEF) regulates differentiation of epithelial cells into mucus-producing cells. The latter cells are critical in the production of MUC5AC and MUC5B 42. Analyzing the expression of these proteins was performed to verify that our lung-on-a-chip accurately replicates key physiological processes, such as mucus production and epithelial cell differentiation, which are essential for proper respiratory function (Fig. 5A). MUC5AC, MUC5B, and SPDEF were substantially expressed in encapsulated cells within the respiratory airway chambers at both the protein (Fig. 5B) and mRNA (Fig. 5C) levels. In lung tissue, the production of glycogen, mucopolysaccharides, and other carbohydrate-rich macromolecules is crucial for evaluating the presence of mucus-producing cells and their functions, which are essential for mimicking the natural lung tissue environment. Periodic acid-Schiff (PAS) staining was performed to evaluate the production and secretion of these components on the lung-on-a-chip. Small airway epithelial cells encapsulated in the respiratory airway chamber of the chip exhibited a uniform PAS-positive reaction, indicating the presence of glycoproteins and proteoglycans typically found in normal lung tissues (Fig. 5D).

Collagen type I (COL1A1) is a major component of the extracellular matrix (ECM) in lung tissue. COL1A1 is pivotal in maintaining the structural integrity of the lungs 43. Fibronectin is another ECM protein that plays key roles in lung cell adhesion, growth, migration, and differentiation 44. Laminin is a vital component of the basement membrane that supports the lung epithelial cell layers and influences their differentiation, migration, and adhesion 45. In this context, the expression of these essential proteins indicated that the lung-on-a-chip appropriately replicates various functional aspects of normal lung tissue (Fig. 5E). COL1A1, fibronectin 1, and laminin α1 were prominently expressed in the encapsulated cells within the supporting stromal chambers at the protein (Fig. 5F) and mRNA (Fig. 5G) levels. Subsequently, ELISA examinations confirmed that fibronectin and laminin were synthesized and secreted from the supporting stromal chamber (Fig. 5H). The findings suggest that the lung-on-a-chip successfully replicates the ECM composition of natural lung tissue.

### Identification and verification of the SERPINB2 biomarker for reliably anticipating lung toxicity in human small airway epithelial cells

**Figure 6:**
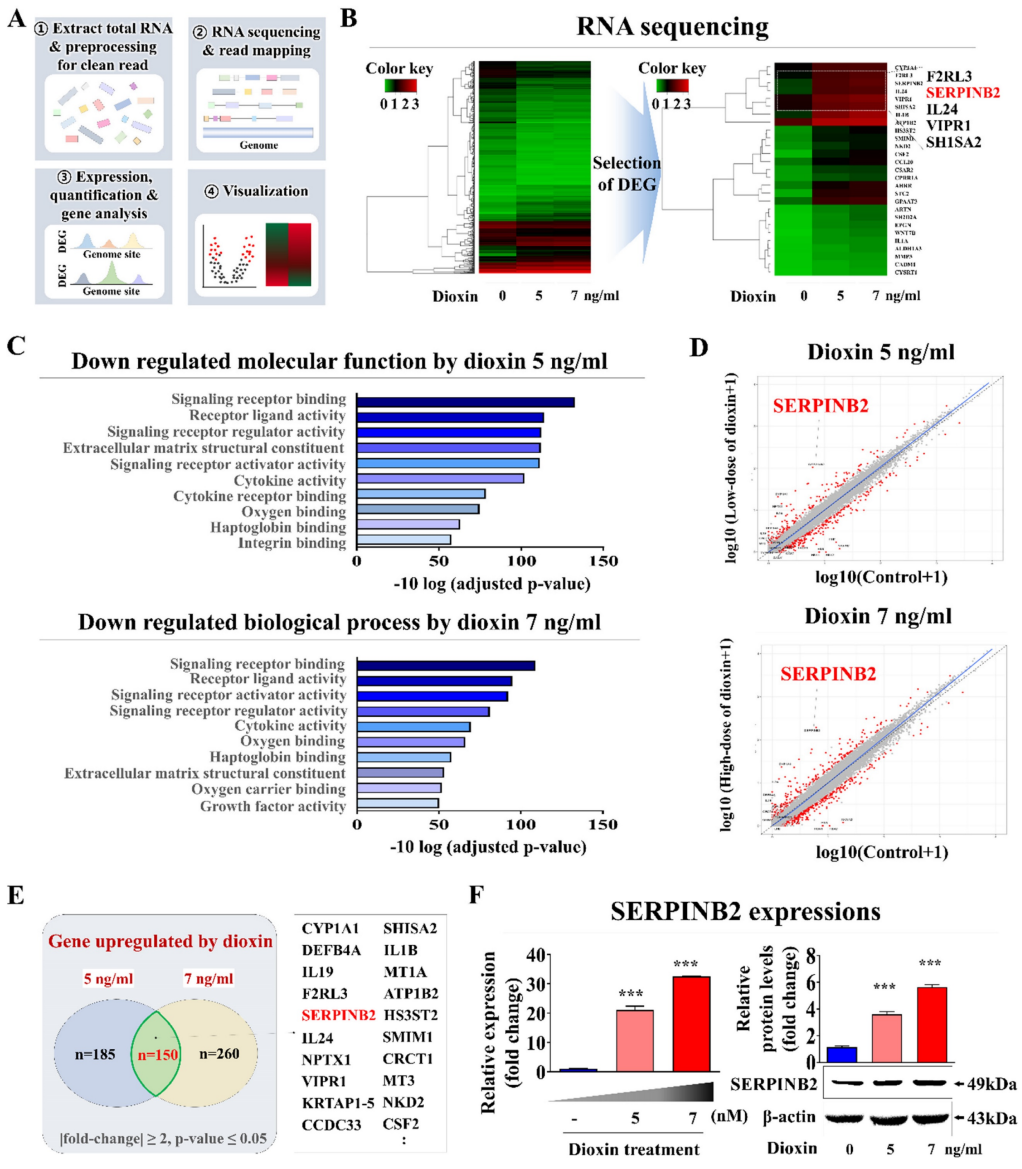
** Discovery and confirmation of a reliable indicator to anticipating lung toxicity in human small airway epithelial cells.** Depiction of the primary stages for the RNA-Seq methodology, comprising the experimental layout, alignment of read, measurement, and graphical representation **(A)**. Comprehensive RNA-seq results displayed in a heatmap that illustrates variations in gene expression between the control groups and those exposed to low (5 ng/ml) and high (7 ng/ml) dioxin doses. The heatmap highlights genes with upregulated (red) or downregulated (green) expression relative to the mRNA levels in the control groups **(B)**. KEGG pathway analyses to identify the potential interconnected pathways and functionalities impacted by toxin exposure **(C)**. Within the array of genes showing differential expression, an observable positive correlation was detected between the marked elevation of SERPINB2 expression and toxic exposure in human small airway epithelial cells **(D and E)**. Using real-time PCR and western blot analysis confirmation of increased SERPINB2 levels following exposure to both low and high doses of the toxin **(F)**. β-Actin was used as the internal protein control, and PPIA was used as the housekeeping gene for real-time PCR. All experiments were performed in triplicate. The data are presented as the means ± SDs. *, *p* < 0.05; **, *p* < 0.005; and ***, *p* < 0.001 (two-sample t-test).

Small airway epithelial cells serve as a first line of defense within the respiratory tract, directly interacting with airborne toxins, pathogens, and particulate matter 46. Their exposure to these substances *in vivo* often results in an immediate cellular response, including inflammatory signaling, oxidative stress, and metabolic disruption, which can serve as early markers of toxicity. This makes small airway epithelial cells a biologically relevant choice for detecting subtle toxicity effects at an early stage, potentially before systemic effects manifest in other lung cell types, such as stromal or endothelial cells. By focusing on these cells, our platform can achieve a rapid, sensitive response to toxic exposures that directly affect the respiratory epithelium. In this context, genome-wide genetic expression profiles were examined in human small airway epithelial cells exposed to a proven toxic material. Fig. 6A presents a schematic overview of the various steps involved in the RNA-seq analysis of human small airway epithelial cells. The toxic compound that was selected was dioxin (2,3,7,8-tetrachlorodibenzo-p-dioxin). It has been identified as a high-priority hazardous chemical by five different global organizations. Numerous genes that showed significantly enhanced expression upon exposure to 5 and 7 ng/ml of standard toxic compounds were identified in human small airway epithelial cells (Fig. 6B). Additionally, Kyoto Encyclopedia of Genes and Genomes (KEGG) pathway examination was performed to assess the biological relationships between the toxic exposure and multiple signaling pathways. Signaling networks associated with cell proliferation and survival, including signaling receptor regulatory activity and cytokine receptor binding, were significantly reduced by toxic exposure (Fig. 6C). Among the genes with elevated expression due to both toxin dosage groups, a notable positive relationship was found between elevated levels of SERPINB2 and hazardous exposure in human small airway epithelial cells (Fig. 6D and 6E). Increased expression of SERPINB2 in response to exposure to toxin was confirmed by quantitative PCR (qPCR) and western blotting. A concentration-dependent response was evident (Fig. 6F). Gene Expression Omnibus (GEO) dataset analysis verified the relationship between elevated SERPINB2 expression and exposure to various toxic substances, including cisplatin, hypochlorous acid, phosgene, trichostatin A, and smoke (Supplementary Fig. 5). As well, human small airway epithelial cells were exposed to various well-established toxic substances, and the expression of the SERPINB2 lung toxicity biomarker in cells was analyzed by qPCR to confirm whether it could serve as a generally applicable indicator for other toxic materials in addition to dioxins. Strongly positive relationships were evident between exposure to a range of established toxicants and elevated SERPINB2 expression in human small airway epithelial cells (Supplementary Fig. 6).

**Figure 7:**
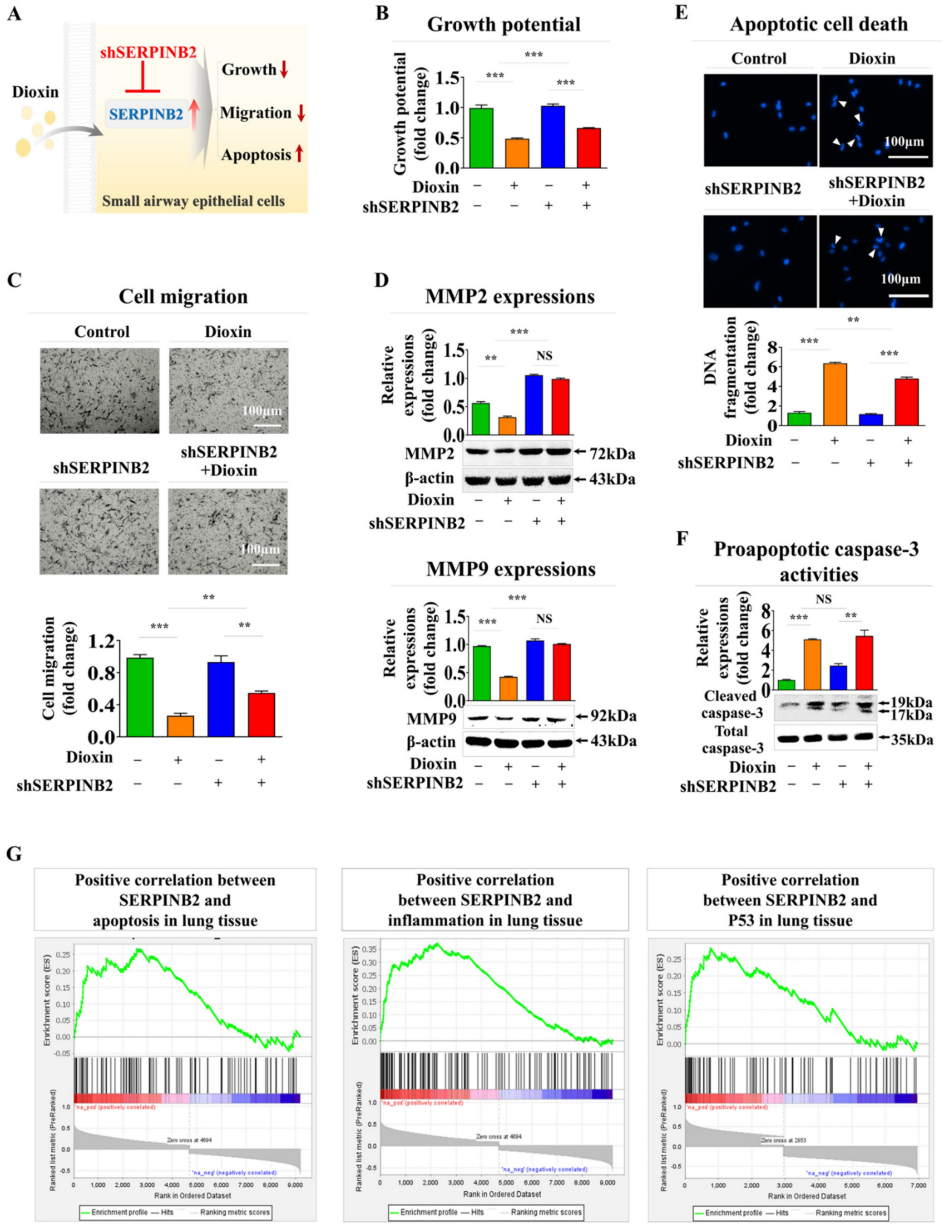
** Validating the consistency of the selected indicator (SERPINB2) for lung toxicity in human small airway epithelial cells.** Diagrammatic representation outlining how SERPINB2 regulates toxicant-triggered detrimental impacts in small airway epithelial cells **(A)**. Human small airway epithelial cells were transfected with a specific SERPINB2 shRNA and were subsequently exposed or not exposed to dioxin (5 ng/ml) for 72 h. The detrimental impacts on cell growth were evaluated through an MTT assay **(B)**. Depleting SERPINB2 effectively neutralized the detrimental effects of dioxin on the migration of small airway epithelial cells. This outcome was confirmed using a Transwell migration/invasion assay **(C)** and western blot analysis with antibodies targeting MMP-2 and MMP-9 **(D)**. Human small airway epithelial cells underwent transfection with a targeted SERPINB2 shRNA and were either exposed or not exposed to 5 ng/ml of dioxin. Following this treatment, alterations in DNA fragmentation associated with apoptosis and caspase-3 mediated apoptotic activities were assessed through nuclear staining **(E)** and western blot analysis **(F)**, respectively. β-actin was used as an internal control. All experiments were performed in triplicate. Data are presented as means ± standard deviations. *, *p* < 0.05; **, *p* < 0.005; and ***, *p* < 0.001 (two-sample t-test).

In addition, SERPINB2 expression was inhibited using a targeted short hairpin RNA (shRNA) against SERPINB2 in human small airway epithelial cells to determine whether SERPINB2 functions as a pivotal regulator of diverse cellular functions linked to toxicity (Supplementary Fig. 7). The effect of SERPINB2 knockdown on different cellular events, including cell death, proliferation, and migration, both in the presence and absence of dioxin, was examined (Fig. 7A). Notably, dioxin-mediated inhibition of cell growth was significantly eliminated by SERPINB2 depletion in human small airway epithelial cells (Fig. 7B). Dioxin-induced suppression of cellular motility (Fig. 7C) and expression of matrix metalloproteinase-2 (MMP-2) and MMP-9 was also markedly abolished by SERPINB2 knockdown in human small airway epithelial cells (Fig. 7D). Dioxin-mediated activation of apoptosis-related DNA breakage (Fig. 7E) and the levels of apoptotic cleaved caspase-3 (Fig. 7F) were mitigated by SERPINB2 knockdown. Furthermore, use of the gene set enrichment analysis algorithm to examine the profiles of differentially expressed genes under diverse pathological states revealed that SERPINB2 expression was significantly elevated in lung tissue affected by various degenerative conditions, such as cell death and inflammation (Fig. 7G).

### Establishment of a luminescent dye-linked toxicity reporter platform predicting lung toxicity

**Figure 8:**
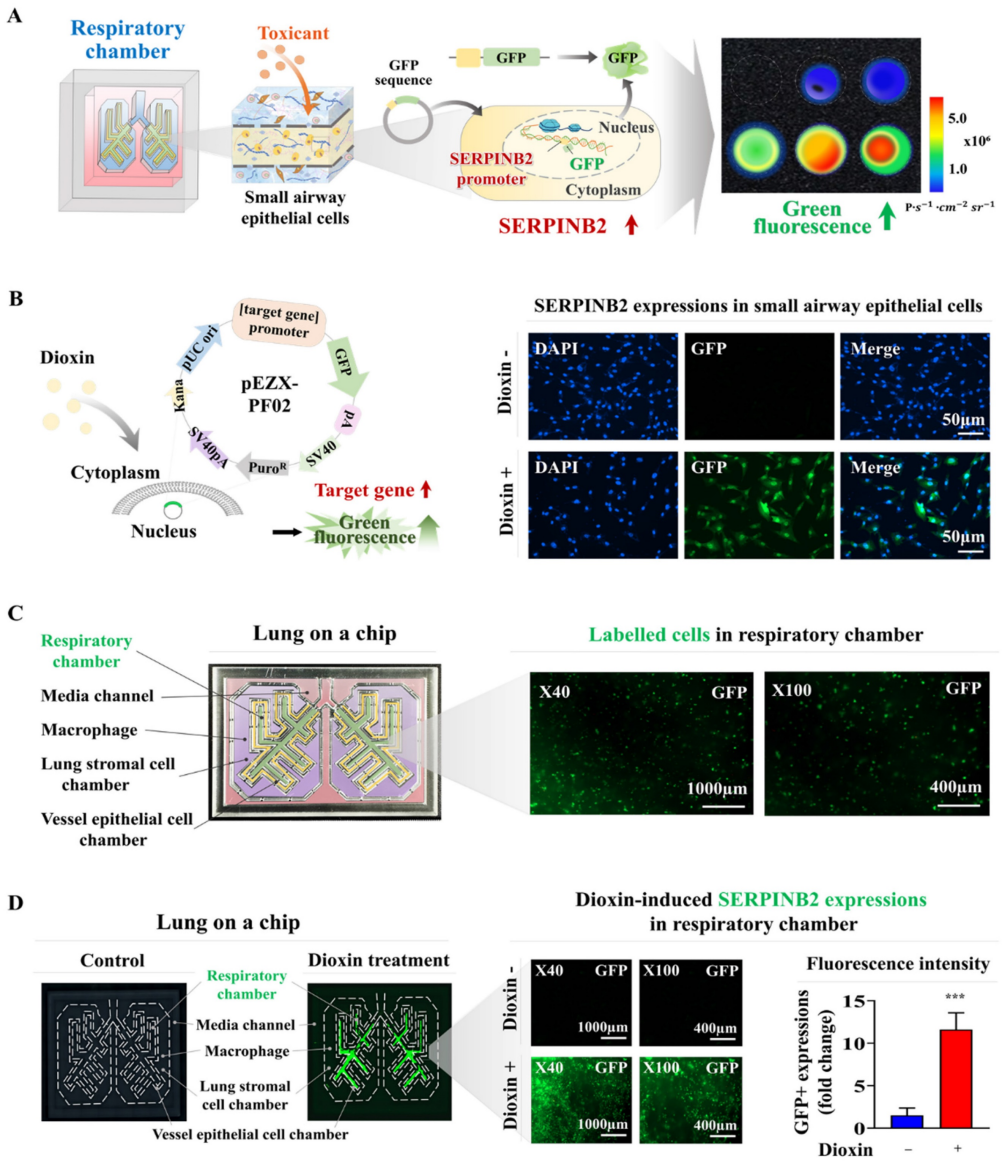
** Development of a fluorescent-based detection platform linked with a toxicity detection marker in the respiratory airway compartment of the lung-on-a-chip model.** A fluorescent detection platform tagged with SERPINB2 was effectively incorporated into human small airway epithelial cells. Toxin-stimulated SERPINB2 activity was evident as fluorescence of into GFP. Consequently, the lung toxicity of specific drug candidates can be assessed in both qualitative and quantitative manner through the measurement of fluorescent signal strength **(A)**. Human small airway epithelial cells underwent stable transfection with a GFP-tagged SERPINB2 detection vector, which emits a green color. Following exposure to 5 ng/ml dioxin, immunostaining indicated a significant increase in SERPINB2 activity, which was manifest as green fluorescence in these cells **(B)**. This fluorescence reporting platform was implemented in human small airway epithelial cells accurately positioned within the respiratory airway compartment of the lung-on-a-chip. The spatial distribution patterns of the incorporated human small airway epithelial cells within respiratory airway chamber then examined through a fluorescent imaging system **(C)**. Exposure to 5 ng/ml dioxin markedly enhanced SERPINB2 activity, which was evident as green fluorescence within the respiratory airway compartment of the chip **(D)**. The data are presented as the means ± standard deviations. *, *p* < 0.05; **, *p* < 0.005; and ***, *p* < 0.001 (two-sample t-test).

**Figure 9:**
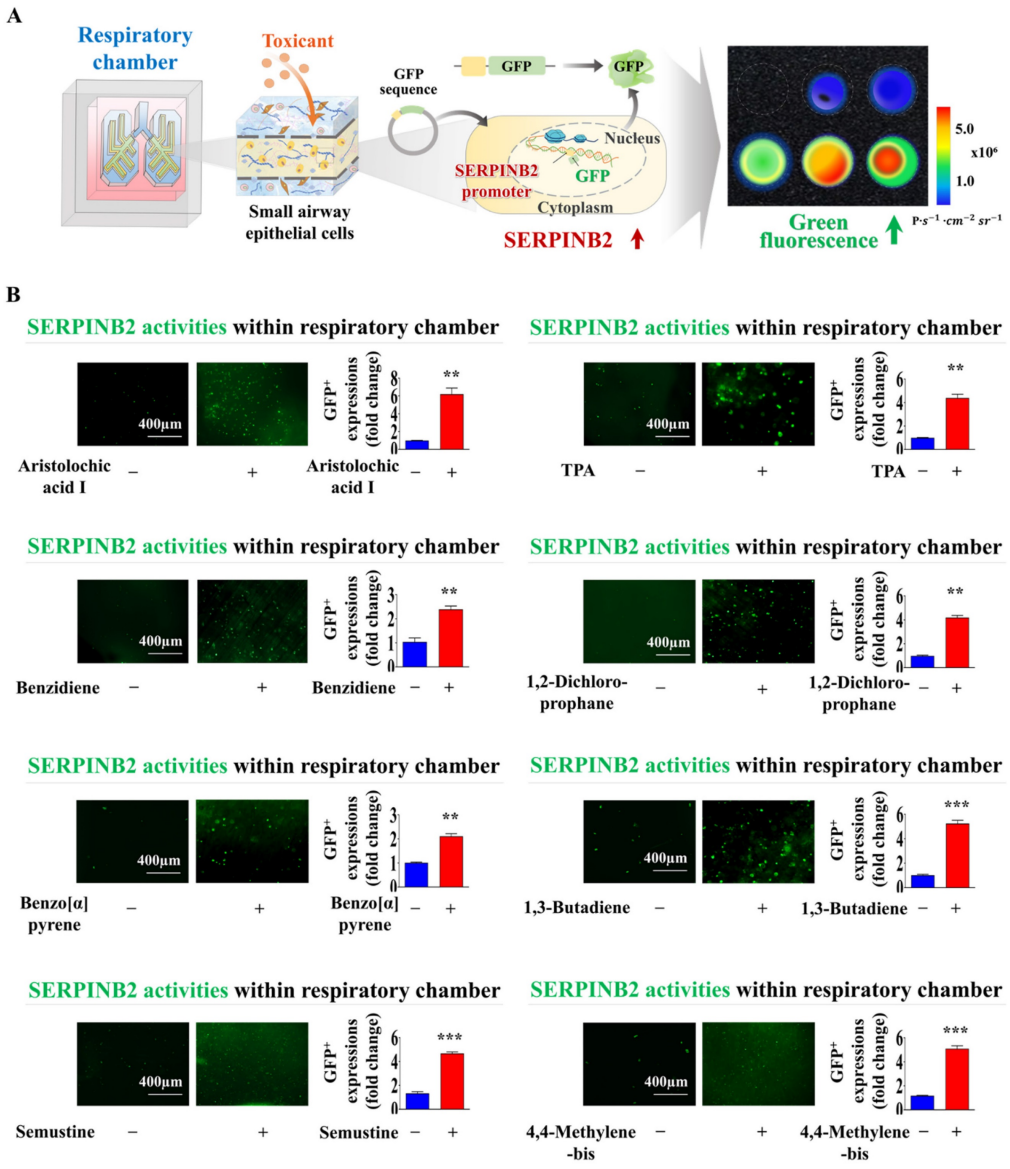
** Exposure to different types of toxins triggers the activation of SERPINB2, resulting in emission of green fluorescence within the respiratory airway chamber of the chip.** A GFP-tagged SERPINB2 fluorescent detection platform was effectively incorporated into human small airway epithelial cells. Subsequently, these cells were positioned in the appropriate compartments of the chip **(A)**. To validate the consistency of the toxicity assessment marker (SERPINB2)-tagged fluorescent detection platform, toxins were added to the respiratory airway chamber of the chip. The toxins included aristolochic acid I (10 μM), benzidine (10 μM), benzo[a]pyrene (2 μM), semustine (0.5 mM), TPA (5 nM), 1,2-dichloropropane (100 mM), 1,3-butadiene (10 mM), and 4,4'-methylenebis (5 μM). The response of SERPINB2 to various types of toxic exposure was assessed both subjectively and quantitatively through the measurement of the resultant fluorescent signal strength **(B)**. Significant differences are indicated as follows: *, *p* < 0.05; **, *p* < 0.005; and ***, *p* < 0.001 (two-sample t-test).

Fluorescent protein-linked screening platforms are extensively employed to assess the activities of specific genes or signaling cascades. These platforms are a robust detection tool for assessing the potential effects and possible adverse effects of drug candidates 47-49. Our findings suggest that SERPINB2 is a robust and widely applicable indicator of lung toxicity. Therefore, GFP was integrated into the promoter region of SERPINB2 in human small airway epithelial cells. The subsequent activation of SERPINB2 by exposure to toxins induces a green fluorescence signal in the lung-on-a-chip. This fluorescent dye-linked toxicity reporter platform enabled the intuitive and quantifiable evaluation of potential lung toxicity based on the fluorescence signal strength (Fig. 8A). Consequently, human small airway epithelial cells transformed with a GFP-linked SERPINB2 reporter construct were integrated into the respiratory airway chamber of the lung-on-a-chip (Fig. 8B). The incorporated cells tagged with GFP were uniformly spread across the respiratory airway chamber of the lung-on-a-chip (Fig. 8C). To evaluate the efficacy of the GFP-linked SERPINB2 detection platform in assessing lung toxicity, SERPINB2 expression levels in the respiratory airway chamber, mediated by dioxin exposure, were determined by quantifying GFP fluorescence (Fig. 8D). To further assess whether the GFP-associated SERPINB2 detection platform could function as a “general” system for evaluating lung toxicity from various categories of toxic substances, rather than serving solely as a specific biomarker of a reference toxin, such as dioxin. GFP fluorescence linked to SERPINB2 activities were quantified in the presence and absence of multiple types of toxic substances in the respiratory airway chambers (Fig. 9A). Every tested toxic substance markedly enhanced SERPINB2-associated GFP fluorescence in the respiratory airway chambers of the lung-on-a-chip (Fig. 9B). These results indicate that the GFP fluorescence-associated SERPINB2 toxicity biomarker-based screening platform is an effective and reliable system for assessing lung toxicity of various potential therapeutic candidates.

## Discussion

Traditional unicellular monolayer culture models are the most commonly employed *in vitro* testing platforms to evaluate the effectiveness and adverse effects of prospective drug candidates. Although these conventional culture models provide advantages, such as rapid setup, simplicity, and flexibility, they fail to properly replicate the complex tissue environment. Specifically, they lack the intricate cell-ECM and cell-cell interactions that are fundamental to tissue function. These critical interactions are essential for maintaining physiological tissue integrity and influencing responses to stimuli 50. Consequently, these cultures occasionally yield unpredictable and misleading results and fail to accurately replicate *in vivo* responses 51. Significant advancements have been made in the development of sophisticated lung-on-a-chip platforms to address the inherent limitations of traditional 2D chip platforms. For example, Zamprogno *et al.* developed a lung-on-a-chip using a biodegradable and flexible membrane composed of elastin and collagen that simulated a series of tiny alveoli with dimensions similar to those found* in vivo* 4. The authors attempted to recreate the lung air-blood barrier by utilizing primary lung endothelial lung alveolar epithelial cells obtained from patient samples. Baptista *et al.* also fabricated a microfluidic lung-on-a-chip device, which consisted of top and bottom cell culture chambers separated by a permeable membrane. Primary human alveolar epithelial cells were infused into microscale curved seed membranes. The top side of these membrane was seeded with Calu-3 lung adenocarcinoma cells, and the bottom side was populated with human lung microvascular endothelial cells 2. Dasgupta *et al.* developed a microfluidic lung alveolus chip lined with human lung alveolar epithelium and interfaced with pulmonary endothelium. This model was designed to simulate acute radiation-induced lung injury in the human lungs *in vitro*. Six hours after radiation exposure, the lung epithelium displayed DNA impairment, hypertrophic growth, increased pro-inflammatory cytokine expression, and compromised barrier function 52.

Although these lung-on-a-chip systems strive to replicate the intricate architecture and dynamic environment of human lungs, they are currently constrained by several significant technical challenges. For instance, accurately replicating the lung tissue requires obtaining and maintaining a diverse array of lung cells, including alveolar epithelial cells, vessel endothelial cells, supporting stromal cells (fibroblasts), and immune cells, such as macrophages. Each cell type has distinct culture requirements and behaviors, making co-culture in a single-chip system complex and prone to variability. Furthermore, the long-term viability and functionality of these incorporated cells within the chip platform remain problematic owing to the lack of a surrounding 3D microenvironment and their intercellular communication. In this context, we developed an innovative lung-on-a-chip 3D culture system. This advanced platform integrates various human lung cell types, such as alveolar epithelial cells, endothelial cells, fibroblasts, and immune cells, with a mixture of biodegradable natural polymers (hyaluronic acid and collagen) and blood coagulation factors (thrombin and fibrinogen). By combining these elements, the system successfully replicates the unique properties and complexities of the pulmonary tissue microenvironment. This integration allows for the precise simulation of multicellular interactions characteristic of complex lung tissues, including dynamic cell-cell and cell-ECM communications. Consequently, it provides optimal *in vivo*-like conditions to ensure long-term cellular viability and functionality. While the cell survival rates gradually declined, the majority of incorporated cells (>87%) were still viable after 14 days (Fig. 3B). When integrated into the natural polymer-based 3D microenvironment, various lung constituent cells expressed their characteristic markers (Fig. 4) and effectively sustained their functions (Fig. 5) over an extended period.

Ensuring the derivation of stable and reproducible test evaluation results is a critical challenge that must be addressed. The variability in outcomes when using primary cultured lung cells is influenced by several factors, including donor variability, passage number, and expertise of the personnel involved in cell isolation and culture. Primary lung cells from different patients can exhibit significant heterogeneity owing to genetic differences, pre-existing health conditions, and environmental exposure, leading to inconsistent responses to experimental conditions. Therefore, to address the limitations associated with primary cultured cells, we immortalized various lung constituent cells using the SV40 large T antigen. These immortalized cells were integrated into an engineered lung-on-a-chip platform. Immortalized cells generally exhibit greater genetic and phenotypic stability over successive passages than primary cells. The use of simian virus 40 (SV40) large T antigen immortalized human cells for organ-on-a-chip development offers substantial advantages over primary cells. These advantages include enhanced proliferation and longevity, improved reproducibility, scalability for high-throughput applications, reduced ethical concerns, and greater stability and genetic manipulability. Immortalized cells were evaluated for the expression of key markers indicative of their tissue-specific functions. Notably, cytokeratins 18 and 19-recognized as markers of small airway epithelial cells-were highly expressed within the incorporated cells (Fig. 4A). Additionally, fibronectin and vimentin, which are essential lung stromal cell proteins involved in cell migration and invasion, were prominently present in the integrated stromal cells (Fig. 4B). The consistent expression of these cell-specific markers suggests that these immortalized cells maintained their intrinsic characteristics (Fig. 4A-D).

Another critical challenge in the successful development of lung-on-a-chip is the establishment of an optimal quantitative measurement system to detect molecular and physiological changes in response to external stimuli. Achieving precise and reliable quantification of these changes is essential for accurately assessing the dynamic responses of lung tissue to various conditions, thereby enhancing the utility and effectiveness of the lung-on-a-chip platform. Specific biomarker (gene)-based screening strategies offer significant advantages over traditional morphological or functional parameter-based approaches, such as those that rely on phenotypic changes, cell growth, or apoptosis. Biomarkers can detect early disturbances in cellular processes, such as oxidative stress, DNA damage, and inflammatory responses, enabling the identification of toxic effects more quickly and sensitively than functional parameter-based methods. This allows the recognition of toxicity before significant cellular damage or death occurs. Therefore, we identified SERPINB2 as a reliable indicator of lung toxicity of specific substances by performing RNA-seq analysis (Fig. 6A) and examining the relationship between exposure to toxic substances and their associated signaling pathways (Fig. 6C and Supplementary Fig 5). In agreement with our findings, previous research has demonstrated a notable upregulation of SERPINB2 expression in human umbilical cord blood stem cells 53, endometrial stem cells 54, and various types of cancer stem cells 55 upon exposure to multiple toxic substances. This toxic substance-induced suppression of diverse cellular activities was markedly reversed by SERPINB2 knockdown in small airway epithelial cells (Fig. 7). These findings indicate that SERPINB2 is a reliable indicator of lung toxicity. Subsequently, GFP was integrated into the upstream regulatory element of SERPINB2 in small airway epithelial cells (Fig. 8B). The GFP-conjugated reporter activity upon exposure to toxic substances was quantitatively assessed by measuring the fluorescent signal strength (Fig. 8D). Moreover, substantially elevated SERPINB2-linked GFP fluorescent signals were detected in small airway epithelial cells following exposure to various toxic substances (Fig. 9). These results highlight the essential prerequisites for establishing a biomarker-driven screening system for assessing the lung toxicity of prospective drug candidates. Our current lung-on-a-chip platform utilizes a modular design, which allows the colorimetric system to be scaled by integrating multiple chips within a single array, each configured with small airway epithelial cells expressing the fluorescent marker SERPINB2. This modular approach can facilitate simultaneous, parallel testing of multiple samples or toxic compounds, addressing a core requirement for HTS systems. Furthermore, our results (Fig. 9, Supplemental Fig. 6) demonstrate that the fluorescent marker system exhibits high sensitivity to a range of toxic compounds, with the capacity to detect minimal changes in toxicity-related biomarkers within a rapid response timeframe. Specifically, the integration of GFP-tagged SERPINB2 within the chip provides a highly sensitive and quantifiable fluorescence signal, which is ideal for distinguishing subtle variations in toxicity levels across different compounds. This sensitivity is a critical advantage, as it enables early-stage detection of toxicity effects prior to cellular morphological changes, making it especially suitable for high-throughput applications where quick readouts are essential. While qPCR provides precise, endpoint quantification of specific biomarkers, the primary advantage of this colorimetric fluorescence approach lies in its capability for real-time, dynamic monitoring of toxicity responses in live cells. The fluorescence intensity associated with the SERPINB2-linked GFP marker serves as a direct indicator of cellular reactions to toxic agents, allowing continuous observation of biomarker activity without disrupting the experimental environment. Unlike qPCR, which relies on endpoint sampling and thus restricts its use in time-course studies, this method enables researchers to capture the onset and progression of toxicity in real-time. This continuous tracking of cellular responses affords insights into early toxic effects well before the appearance of terminal functional outcomes, advancing our ability to monitor toxicity dynamics with heightened sensitivity and temporal resolution.

Although initially optimized for toxicity assessment using biomarker-linked fluorescence, the platform is highly versatile and can also be tailored to assess the therapeutic potential of drug candidates, making it a multifunctional asset within the drug development process. The modular structure of our lung-on-a-chip system permits adjustments to support therapeutic efficacy studies. By customizing the biomarker systems, we can focus on alternative pathways and cellular responses relevant to therapeutic activity, such as anti-inflammatory effects, tissue repair, or specific molecular mechanisms related to lung health. This adaptability is particularly advantageous for evaluating respiratory drug candidates, as the 3D multicellular lung model, initially designed to monitor toxicity, can also be utilized to observe drug effects aimed at alleviating lung damage or disease progression.

Our current lung-on-a-chip system is designed using normal lung cells; however, the platform's inherent versatility allows for straightforward modifications to simulate a variety of lung pathologies. By incorporating cells derived from diseased tissues or by introducing specific pathological stimuli, this system can be customized to replicate multiple disease states, including but not limited to lung cancers, pulmonary fibrosis, and various inflammatory lung conditions. This adaptability not only enhances the physiological relevance of the platform but also extends its utility to more clinically pertinent applications. For instance, the ability to simulate disease-specific microenvironments allows researchers to conduct detailed studies on the underlying mechanisms of various lung diseases, gaining insights that would be challenging to capture in traditional models. Moreover, by assessing drug responses in these disease-specific settings, the platform offers a valuable tool for evaluating therapeutic efficacy, paving the way for more targeted drug testing and even personalized medicine approaches. This expanded capability makes our lung-on-a-chip a powerful model for both basic research and translational applications, bridging the gap between *in vitro* studies and clinical relevance.

## Supplementary Material

Supplementary figures.

## Figures and Tables

**Table 1:** Primer sequences for quantitative RT-PCR.

Gene	GenBank No.	Direction	Primer sequence
Human PPIA	NM_021130	F	TGCCATCGCCAAGGAGTAG
		R	TGCACAGACGGTCACTCAAA
Human SERPINB2	NM_001143818	F	ACCCCCATGACTCCAGAGAACT
		R	GAGAGCGGAAGGATGAATGGAT
Human COL1A1	NM_ 000088	F	GATTCCCTGGACCTAAAGGTGC
		R	AGCCTCTCCATCTTTGCCAGCA
Human FN1 NM_ 212482		F	ACAACACCGAGGTGACTGAGAC
		R	GGACACAACGATGCTTCCTGAG
Human LAMA1 NM_ 005559		F	GAAGGTGACTGGCTCAGCAAGT
		R	AGGCGTCACAACGGAAATCGTG
Human MUC5AC NM_ 017511		F	CCACTGGTTCTATGGCAACACC
		R	GCCGAAGTCCAGGCTGTGCG
Human MUC5B NM_ 002458		F	CTGCTACGACAAGGACGGAAAC
		R	AAGGCTGTGAGCGCACTGGATG
Human SPDEF NM_012391		F	CGAAGTGCTCAAGGACATCGAG
		R	CGGTATTGGTGCTCTGTCCACA

## Abbreviations

3D: three-dimensional
CAD: computer-aided design
CCK-8: cell cunting kit - 8
DMEM: dulbecco's modified Eagle's medium
ECM: extracellular matrix
FBS: fetal bovine serum
GEO: gene expression omnibus
GFP: green fluorescent protein
HUVECs: Human umbilical vein endothelial cells
KEGG: Kyoyo Encyclopedia of Genes and genomes
KLF4: krüppel-like factor 4
MMP-2/9: matrix metalloproteinase-2
PDMS: polydimethylsiloxane
PLA: polylactic acid
SEM: scanning electron microscopy
SERPINB2: plasminogen activator inhibitor-2
SOX2: sex-determining region Y-box 2
TBS-T: tris-buffered saline containing Tween-20
