# Supplementary material for: Advanced Lung-on-a-Chip Technology: Mimicking the Complex Human Lung Microenvironment
Source: Int J Biol Sci. 2025 Jan 1;21(1):17–39. doi: 10.7150/ijbs.105702 (PMC11667821; doi:10.7150/ijbs.105702)
Supplement: Supplementary file 1 — Supplementary figures. [file ijbsv21p0017s1.pdf]

Supplementary Figures and Legends

Supplementary figure 1

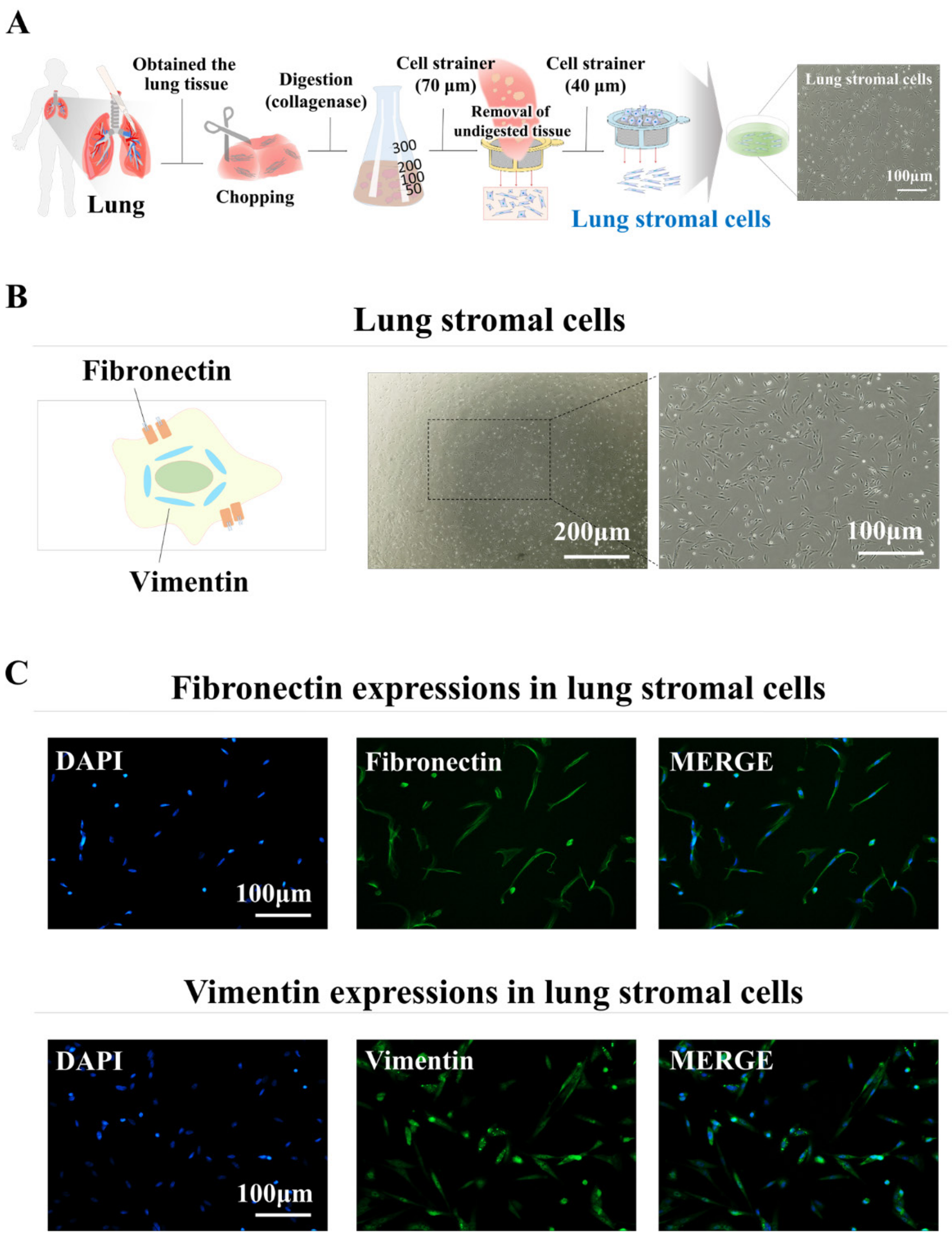

Supplementary Fig. 1. Isolation and molecular properties of isolated human lung stromal cells. Diagram detailing the methods used to isolate human lung stromal cells from patients treated with pneumonectomy or lobectomy for cancer. The isolation involved separating lung stromal cells from other cell types in lung tissue biopsies by passing them through cell strainers with differing pore sizes

(A). Optical phase microscopy revealing the spindle-shaped morphology of human lung stromal cells **(B)**. The expressions of established biomarkers in lung stromal cells, including fibronectin and vimentin, were assessed through immunohistochemical staining **(C)**. DAPI staining was used to label the nuclei within each field.

Supplementary figure 2

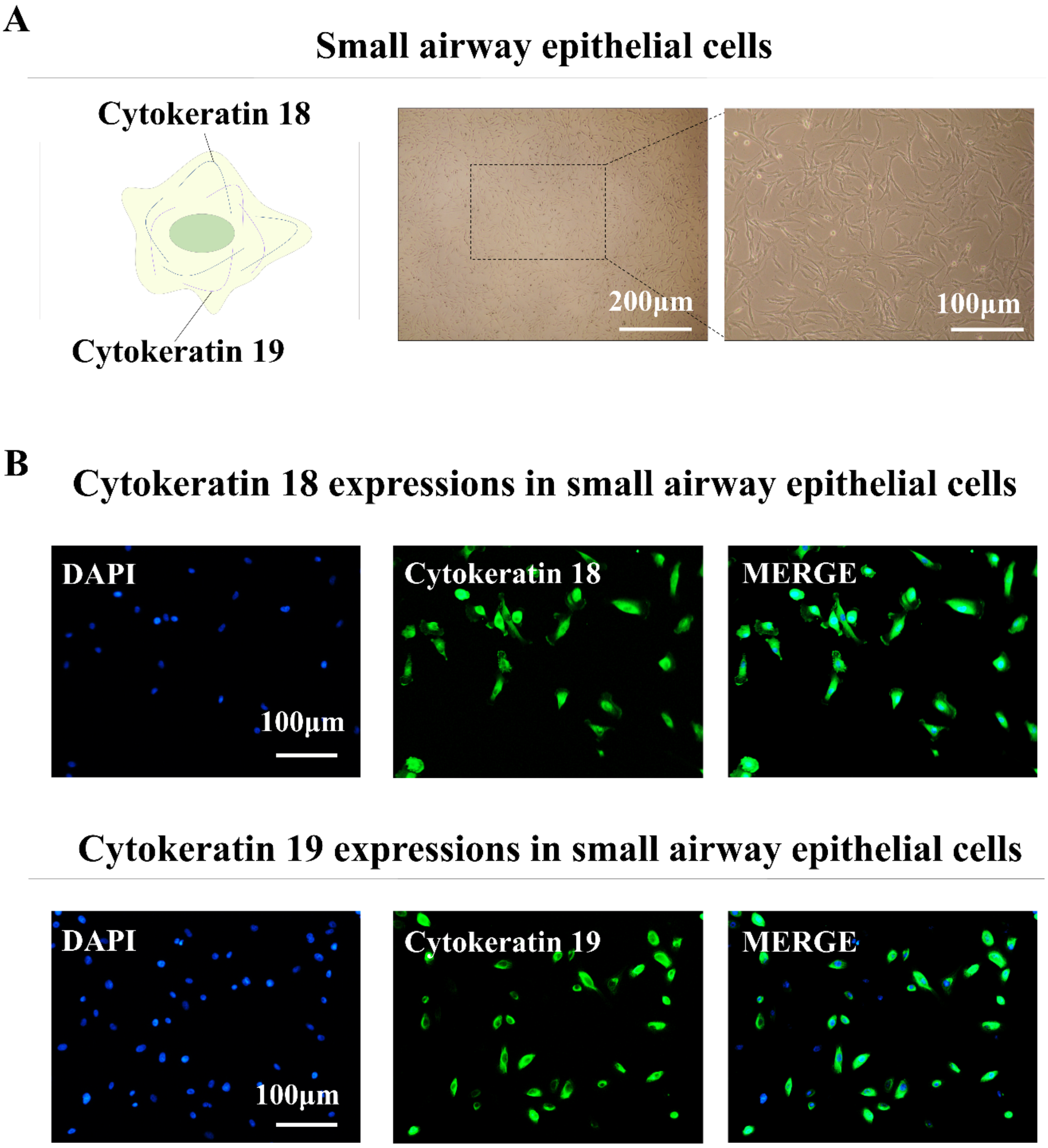

Supplementary Fig. 2. Molecular characterization of human small airway epithelial cells. Small airway epithelial cells were morphologically characterized by their distinct polygonal shape **(A)**. The expression profiles of potential biomarkers cytokeratin 18 and 19 in human small airway epithelial cells were analyzed by using fluorescent immunostaining **(B)**. DAPI staining was used to label the nuclei within each field.

Supplementary figure 3

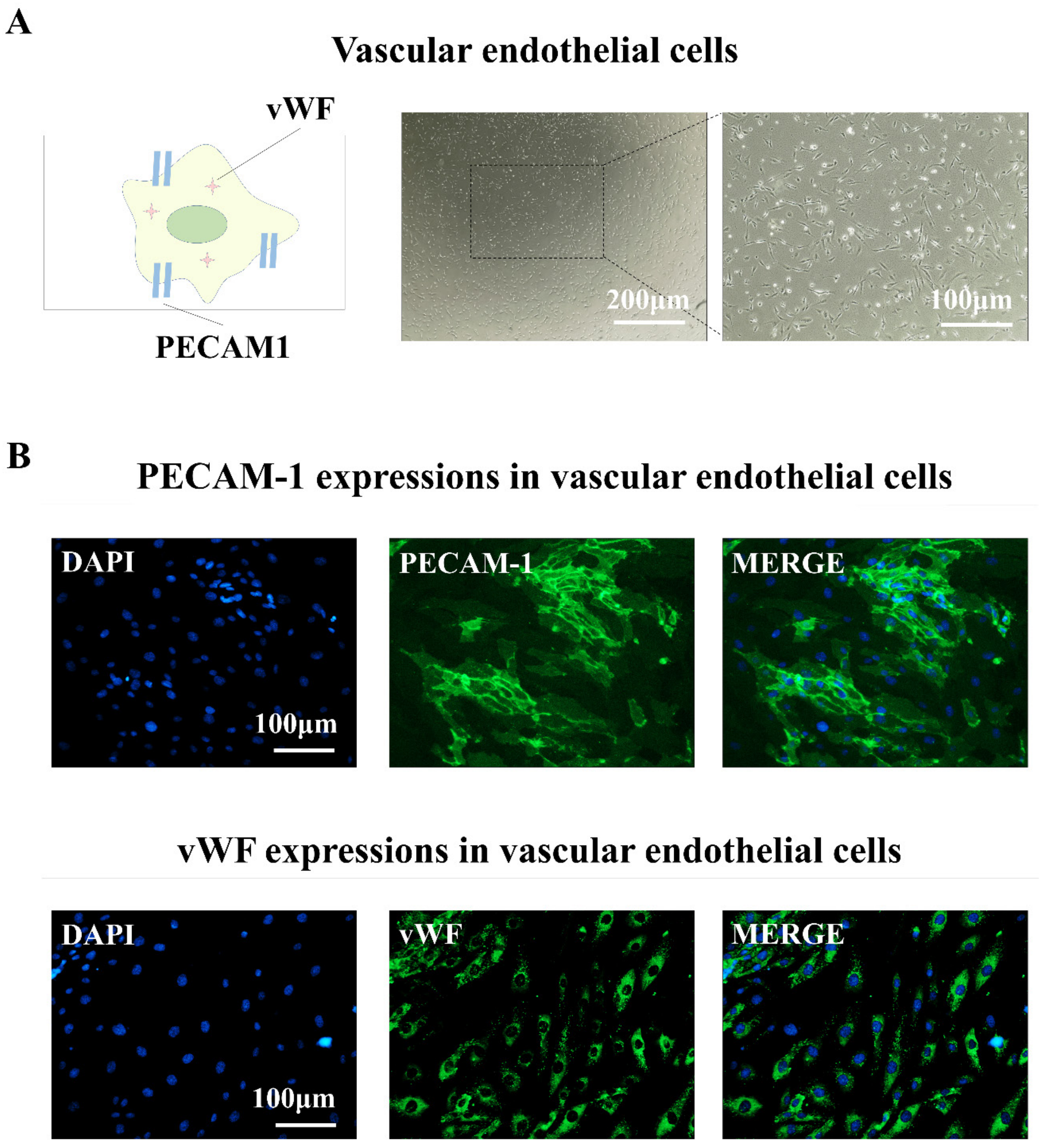

Supplementary Fig. 3. Molecular profiling of human vascular endothelial cells (HUVECs). HUVECs were cultured in a monolayer formation. Phase-contrast microscopy revealed their distinctive polygonal morphology **(A)**. The expression profiles of potential endothelial cell-specific markers, platelet endothelial cell adhesion molecule-1 (PECAM-1) and von Willebrand Factor (vWF), were

assessed using fluorescent immunostaining **(B)**. DAPI staining was used to label the nuclei within each field.

Supplementary figure 4

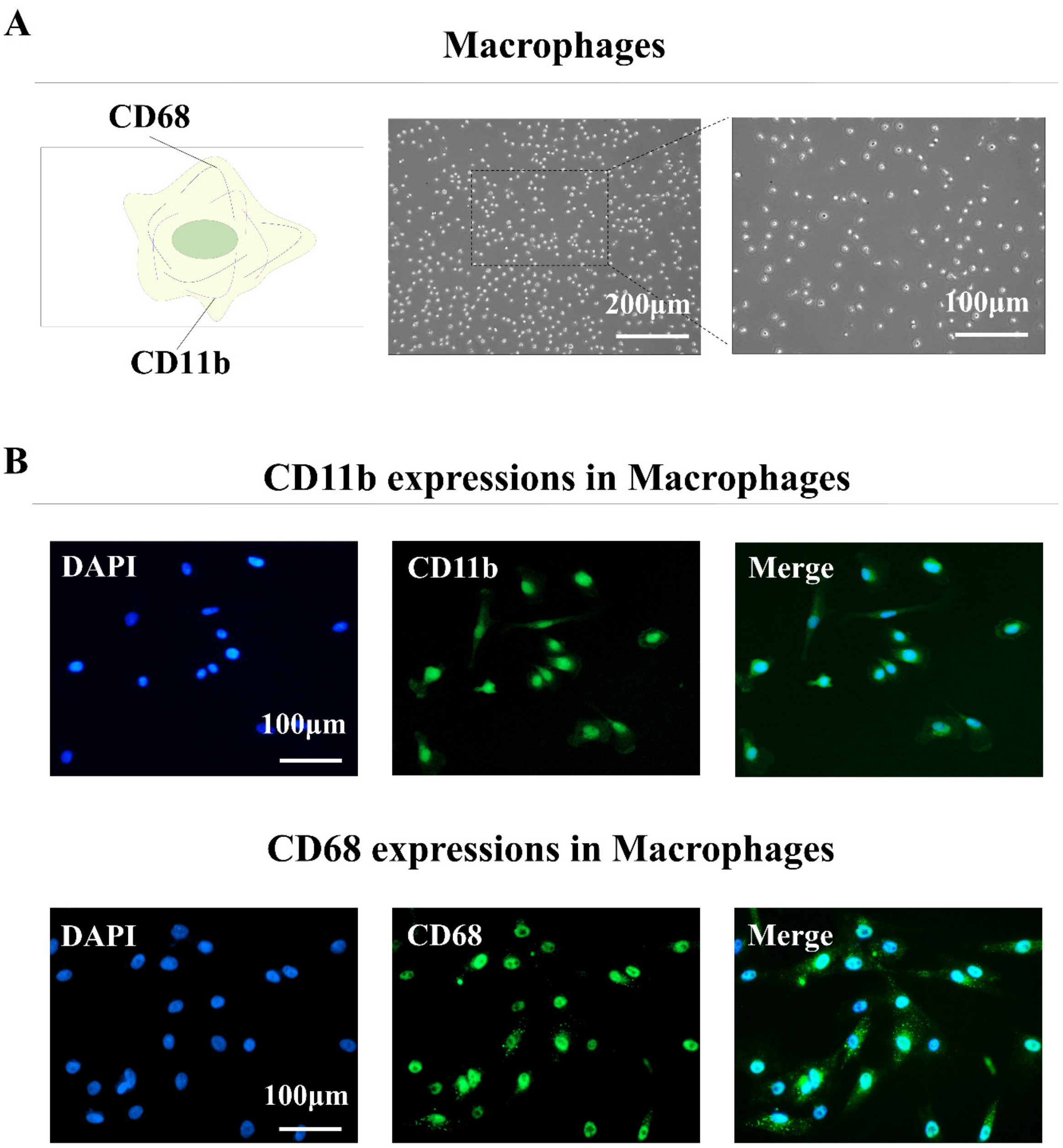

Supplementary Fig. 4. Molecular characterization of human macrophages. Spherical human macrophages were grown as a single-layer culture and examined using phase-contrast microscopy **(A)**. The expression profiles of potential markers CD11b and CD68 in macrophages were analyzed through fluorescent immunostaining **(B)**. DAPI staining was used to label the nuclei within each field.

Supplementary figure 5

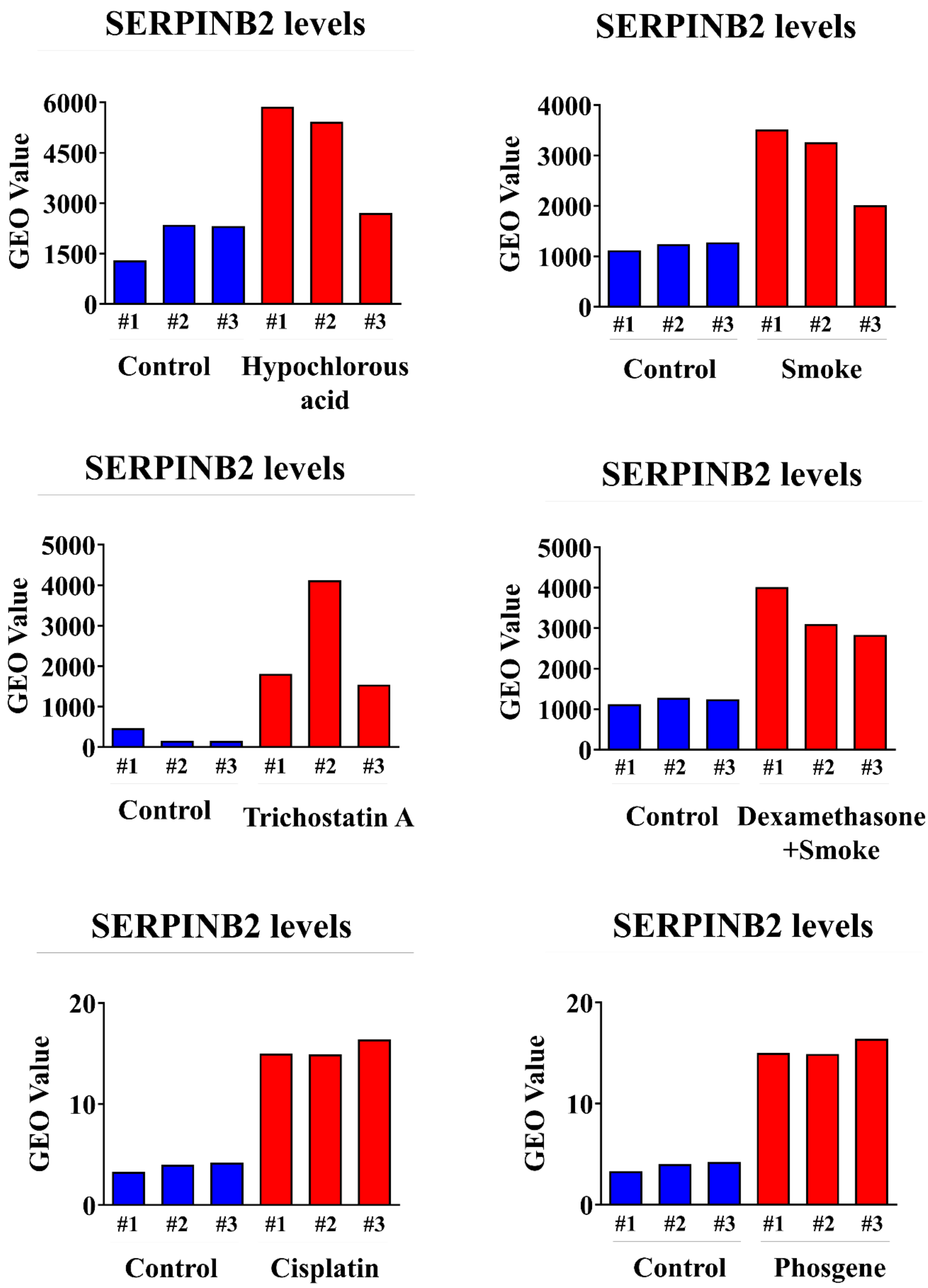

Supplementary Fig. 5. SERPINB2 levels increase significantly in the presence of toxins. Data from the Gene Expression Omnibus (GEO) public database were examined to assess the strong associations between elevated SERPINB2 levels and exposure to various toxic substances, including cisplatin, hypochlorous acid, phosgene, smoke, and trichostatin A.

Supplementary figure 6

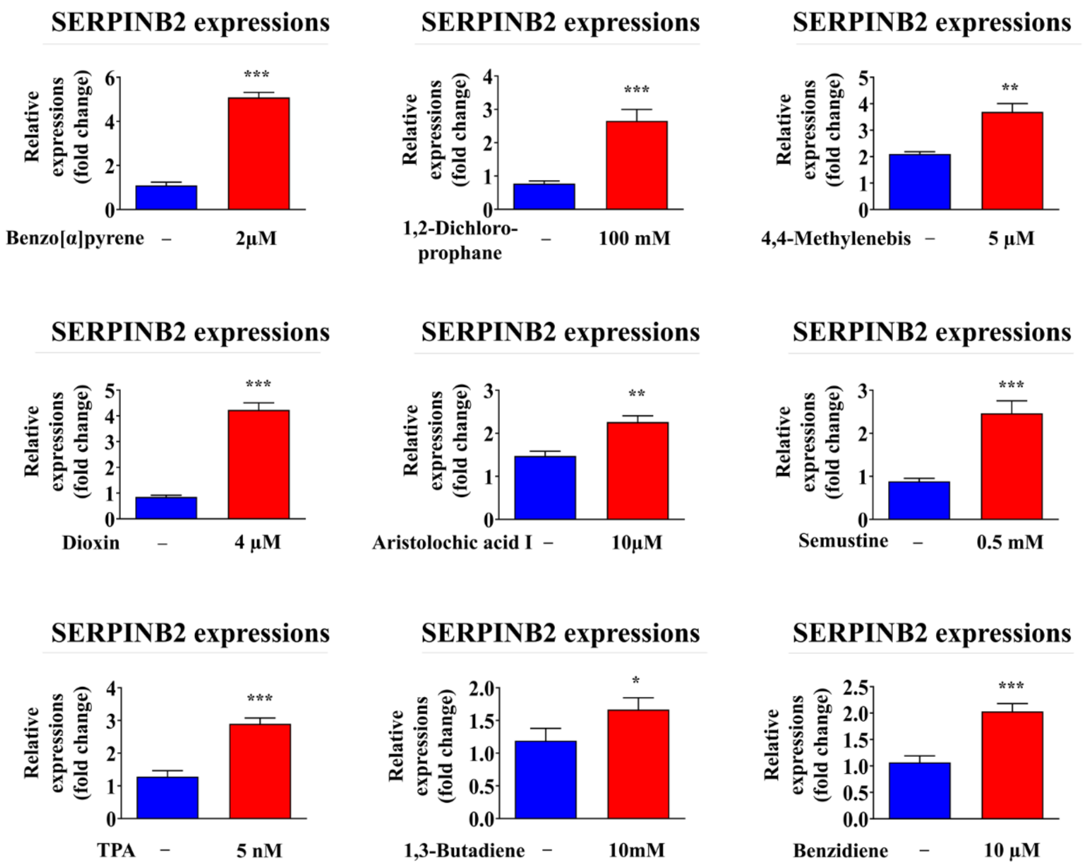

Supplementary Fig. 6. Diverse toxic exposures markedly elevate SERPINB2 expression in human small airway epithelial cells. Real-time PCR was used to determine the SERPINB2 expression levels in human small airway epithelial cells after 72 h of exposure to various toxins, including benzo[a]pyrene (2 μM), 1,2-dichloropropane (100 mM), 4,4’-methylenebis (5 μM), dioxin (5 ng/ml), aristolochic acid I (10 μM), semustine (0.5 mM), TPA (5 nM), 1,3-butadiene (10 mM), and benzidine (10 μM). *PPIA* was used as a housekeeping gene for real-time PCR. Significant differences are presented. *, $p < 0.05$; **, $p < 0.005$; and ***, $p < 0.001$ (two-sample t-test).

Supplementary figure 7

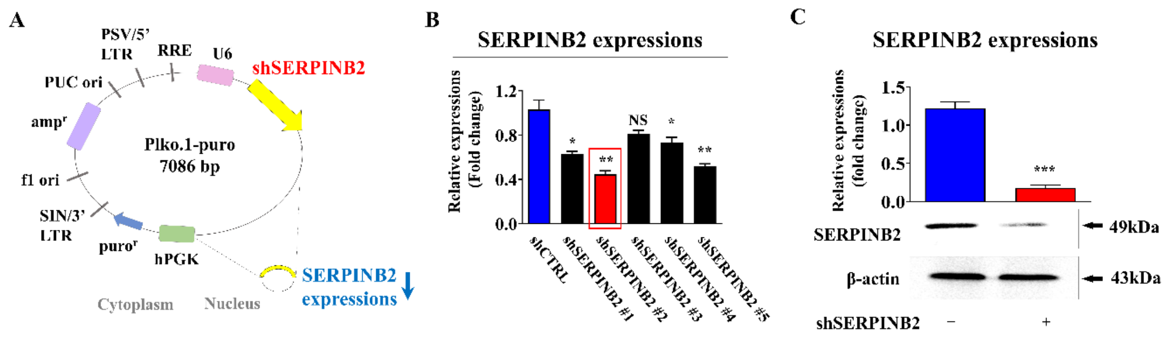

Supplementary Fig. 7. Effectiveness of SERPINB2 suppression via targeted shRNAs in human small airway epithelial cells. Human small airway epithelial cells were effectively transfected with multiple shRNA constructs (designated as #1, #2, #3, #4, or #5), each specifically designed to target SERPINB2 **(A)**. Among the shRNA constructs, #2 (subsequently referred to as SERPINB2 shRNA) was the most effective in reducing SERPINB2 expression, impacting both mRNA **(B)** and protein **(C)** levels. β-actin was used as an internal protein control. *PPIA* was used as a housekeeping gene for real-time PCR. All experiments were performed in triplicate. Data are presented as means ± standard deviations. *, $p < 0.05$; **, $p < 0.005$; and ***, $p < 0.001$ (two-sample t-test).
